# Establishing an infrastructure for collaboration in primate cognition research

**DOI:** 10.1371/journal.pone.0223675

**Published:** 2019-10-24

**Authors:** Many Primates, Drew M. Altschul, Michael J. Beran, Manuel Bohn, Josep Call, Sarah DeTroy, Shona J. Duguid, Crystal L. Egelkamp, Claudia Fichtel, Julia Fischer, Molly Flessert, Daniel Hanus, Daniel B. M. Haun, Lou M. Haux, R. Adriana Hernandez-Aguilar, Esther Herrmann, Lydia M. Hopper, Marine Joly, Fumihiro Kano, Stefanie Keupp, Alicia P. Melis, Alba Motes Rodrigo, Stephen R. Ross, Alejandro Sánchez-Amaro, Yutaro Sato, Vanessa Schmitt, Manon K. Schweinfurth, Amanda M. Seed, Derry Taylor, Christoph J. Völter, Elizabeth Warren, Julia Watzek

**Affiliations:** 1 The University of Edinburgh, Edinburgh, United Kingdom; 2 Georgia State University, Atlanta, Georgia, United States of America; 3 Stanford University, Stanford, California, United States of America; 4 Leipzig University, Leipzig, Germany; 5 University of St Andrews, St Andrews, United Kingdom; 6 Warwick Business School, University of Warwick, Coventry, United Kingdom; 7 Lincoln Park Zoo, Chicago, Illinois, United States of America; 8 German Primate Center and Leibniz ScienceCampus Primate Cognition, Göttingen, Germany; 9 Max Planck Institute for Evolutionary Anthropology, Leipzig, Germany; 10 Center for Adaptive Rationality, Max Planck Institute for Human Development, Berlin, Germany; 11 Centre for Ecological and Evolutionary Synthesis, University of Oslo, Oslo, Norway; 12 Department of Social Psychology and Quantitative Psychology, University of Barcelona, Barcelona, Spain; 13 University of Portsmouth, Portsmouth, United Kingdom; 14 Kyoto University, Kyoto, Japan; 15 University of Tübigen, Tübingen, Germany; 16 University of California San Diego, San Diego, California, United States of America; 17 Heidelberg Zoo & University of Heidelberg, Heidelberg, Germany; 18 Messerli Research Institute, University of Veterinary Medicine Vienna, Medical University of Vienna, University of Vienna, Vienna, Austria; Universidade de São paulo, BRAZIL

## Abstract

Inferring the evolutionary history of cognitive abilities requires large and diverse samples. However, such samples are often beyond the reach of individual researchers or institutions, and studies are often limited to small numbers of species. Consequently, methodological and site-specific-differences across studies can limit comparisons between species. Here we introduce the *ManyPrimates* project, which addresses these challenges by providing a large-scale collaborative framework for comparative studies in primate cognition. To demonstrate the viability of the project we conducted a case study of short-term memory. In this initial study, we were able to include 176 individuals from 12 primate species housed at 11 sites across Africa, Asia, North America and Europe. All subjects were tested in a delayed-response task using consistent methodology across sites. Individuals could access food rewards by remembering the position of the hidden reward after a 0, 15, or 30-second delay. Overall, individuals performed better with shorter delays, as predicted by previous studies. Phylogenetic analysis revealed a strong phylogenetic signal for short-term memory. Although, with only 12 species, the validity of this analysis is limited, our initial results demonstrate the feasibility of a large, collaborative open-science project. We present the *ManyPrimates* project as an exciting opportunity to address open questions in primate cognition and behaviour with large, diverse datasets.

## Introduction

Evolutionary history is a pattern of complex, non-replicable past changes that have led to life as we know it. As a process embedded in time, evolutionary history is inherently opaque. One way to clarify this process is to apply the comparative method; by comparing extant species and their phylogenetic relationship, one can make inferences about the evolutionary history of traits [1]. In addition, quantifying traits in multiple related species allows researchers to link trait variability to the species’ social and/or ecological factors—a key component necessary to draw inferences about the pressures driving the evolutionary process. Because cognitive abilities leave no direct fossil record, the comparative method is especially vital to understanding cognitive evolution. Comparative psychologists have therefore embraced this method in their attempt to elucidate the evolutionary origins of behaviour and cognition. However, the research programme of comparative psychology has not escaped criticism. Notably, comparative psychology has famously been criticised for failing to embrace a sufficient variety of species [2] and to make comparisons between them that adequately reflect their shared ancestry [3]. These limitations undermine confidence in the evolutionary claims made in comparative psychology [4]. Access to diverse samples and techniques to make adequate comparisons between them is therefore essential to the aims of comparative psychology. This approach, faces several challenges. Most captive facilities, where much comparative cognition research is conducted, house only a few species, and often a small number of individual animals per species. The same is true at individual field sites. Increasing both the number and diversity of subjects included in studies requires a new approach: one that is collaborative across researchers and institutions.

Comparative psychologists who study primates do so in diverse settings, including zoos, sanctuaries, laboratories, and in the wild. Both experimental and observational methods can be used in these settings, and there are different costs and benefits associated with each. For example, captive primates can be accessed regularly and are able to take part in controlled cognitive testing. However, since primates have particularly slow life histories [5], captive subjects often participate in many different studies in a lifetime. Thus, site-specific histories of participation in cognitive testing may influence individuals’ task performance [6], an effect that is likely exacerbated by the limited number of individuals housed at each facility, and the tendency for certain facilities’ subjects to be over-represented in the literature (e.g. great ape subjects from the Wolfgang Köhler Primate Research Center in Zoo Leipzig, Germany, are represented in about 250 peer-reviewed studies published between 2006–2016, see [7]). By contrast, cognitive testing is more challenging and often less controlled in wild populations. Wild primates, however, are arguably more representative of their free-living ancestors, meaning field experiments and observations offer indispensable insights into the evolution of primate cognition and behaviour [8]. For a richer perspective on primate cognition, researchers should test multiple species, each living in multiple settings. Yet, despite the benefits of studying primates in diverse settings, the methods used across different sites and species to measure the same trait are often not comparable [9,10]. Often, such differences hinder a quantitative assessment of between- species variability, reduce the likelihood of replicability across sites [6], and limit the inferences that can be made about primate cognitive evolution [9,10]. To ensure a truly comparative approach suited for tackling the key outstanding questions about how cognition evolves, we propose that researchers work together to develop appropriate methods for wide application across species and settings, potentially via large multi-site collaborations.

In addition to the weakness of currently-applied methodological approaches to studying comparative psychology, the analytical approaches used to date also have not been sufficiently sophisticated to adequately compare species’ responses to test paradigms. Typically, researchers make inferences about primate cognitive evolution through direct comparisons of cognitive measurements across species [11–13]. Using this approach, many comparative facts of primate cognition have been established (e.g. [14]). However, the shared evolutionary history between species is seldom considered quantitatively. There is a positive relationship between the amount of shared evolutionary history between species, and their phenotypic similarities [1]. Thus, failing to quantitatively account for shared evolutionary history in cross-species comparisons can give rise to both an over- and under-estimation of species differences [15]. A wealth of statistical methods are now available that allow researchers to account for shared evolutionary history and to examine the evolutionary history of traits by estimating ancestral states, rates of evolutionary change, and making quantitative predictions about species similarities. These are known as phylogenetic comparative methods and they have already been utilized to test possible scenarios regarding the evolution of inhibitory control [11], language [16] and general intelligence [17] within the primate clade.

However, sample size and diversity are key barriers to fully embracing these methods. For instance, Freckleton and colleagues [18] reported that phylogenetic analyses with fewer than 20 species tend to perform poorly—a number much larger than is typical for research in comparative psychology. Moreover, to accurately reconstruct an ancestral state from extant species, samples that reflect the scale of natural diversity in the trait of interest are essential [15]. Some researchers have created primate cognition datasets that are adequate for phylogenetic testing by combining data from different studies and performing meta-analyses [17,19]. However, given the aforementioned current difficulties in interpreting data from different sites and species, meta-analytic approaches to cross-species comparisons are likely to confound species, rearing history, and methodology. Furthermore, meta-analyses are subject to publication bias (leading to overestimated effect sizes) and do not allow for targeting specific species. An alternative approach, as a complement to meta-analyses, is a large-scale collaboration between research groups with a stated goal of data collection, not simply data sharing. In this way, researchers could collect large and diverse samples using agreed-upon methods which are suitably adapted to each study species but are also sufficiently similar to allow for direct comparison across species. Such large-scale collaboration would provide a platform for advancing research in primate cognition and offer an opportunity to develop an infrastructure for replicating findings, sharing expertise, and regulating scientific practice through pre-registration and a shared ethical code, which have been recently highlighted as key limitations of human [20–23] and animal [6] cognition research more generally. The *ManyPrimates* Project was developed to achieve these goals.

### The *ManyPrimates* project

The goal of the *ManyPrimates* project is to facilitate collaboration among primate cognition researchers all over the world, working in a variety of settings. Pooling resources across study sites makes it possible to collect data from samples that are large and diverse enough to tackle questions about the phylogeny, ontogeny and internal structure of primate cognition. A number of previous one-off projects managed to achieve such a pooling of resources [11–13,24–27] demonstrating that such large-scale collaborations are feasible. However, these previous initiatives did not establish a long-lasting infrastructure for future studies to build on. They were mostly initiated and managed by a single individual, focused on a single topic, and relied on dedicated external funding. In contrast, *ManyPrimates* attempts to build an enduring infrastructure for collaboration that is maintained by a diverse group of people, allows the study of a wide range of topics and species, and is not crucially dependent on external funding (for more details on the infrastructure see the Discussion section). In addition, a collaborative project involving a large number of researchers in the field also has the potential to set and promote best practises for research and demonstrate how they can be implemented in a range of settings. From its outset, the *ManyPrimates* project aimed to promote openness, transparency and reproducibility in primate cognition research. In this spirit, all *ManyPrimates* projects will be pre-registered prior to data collection (see https://osf.io/tr6q3/). Although this approach is novel for the field of primatology, such collaborative platforms have been initiated in other fields in psychology, for example the *ManyBabies* project [21], the *Many Labs* project [22], or the Psychological Science Accelerator [23].

The success of such a large-scale project crucially depends on the willingness of people in the field to contribute to it. While many would agree that an infrastructure for large-scale collaboration would greatly benefit the study of primate cognition, investing time and resources in establishing it might appear risky. To generate buy-in, and to evaluate the feasibility of this effort, *ManyPrimates* was inaugurated with a pilot study, which is the empirical focus of this article.

### Short-term memory in primates: A *ManyPrimates* project pilot study

The main empirical goal of the *ManyPrimates* project pilot study was to establish and test an infrastructure for subsequent projects to build on (i.e., to evaluate if an international collaboration was feasible and if a single methodology could be applied by different researchers across species and settings). As a form of validation, we sought to replicate previously-published methods and findings and to expand upon them by estimating the variability across sites and species. To decide on the topic of the pilot study we first collected ideas from early participants in the *ManyPrimates* project. Next, we conducted an online poll based to determine the most popular topic. The results of this poll revealed short-term memory (STM) as our first study topic, tested with a popular methodology (described below).

STM refers to an individual’s ability to retain information for several seconds without memory rehearsal. In contrast to working memory (WM), STM refers to the retention, but not manipulation, of information. In humans, STM has been distinguished empirically from WM in latent variable analyses of individual differences in task performance [28–30] and in investigations of neural correlates [31–35]. Nevertheless, the two terms are often used interchangeably in the literature. Importantly for our purposes, this is a topic that has garnered much interest within the field of comparative psychology, but is one that had not yet been tested with large scale phylogenetic comparative methods.

A common paradigm used for the investigation of primates’ STM is the delayed-response task [36,37]. In this task, subjects watch an experimenter hide a piece of food under one of multiple opaque cups (or behind a screen). After a retention interval, the subject is then given the opportunity to retrieve the food, by choosing between the hiding locations. To obtain the food item, the subject must remember the location of the food reward throughout the retention interval and select only that correct location. The hiding location of the reward is changed from trial to trial, which requires subjects to update their memory between trials. Monkeys and apes are capable of solving these tasks [36–38], and a study by Harlow et al. [39] showed an increased ability to remember correctly progressing from lemurs and South American monkeys to anthropoid apes. Crucially, Harlow et al. noted that there were also within-species individual differences in performance as well as high test-retest correlations, suggesting that short-term memory was a stable capacity of the individual but also one that could highlight species’ and individuals’ differences in a basic cognitive competence. Other comparative studies have found similarities and differences in the STM performance between monkeys, apes, and humans [38, 40]. For example, there is consistent evidence of a decline in performance with increasing number of items that need to be remembered in rhesus macaques (*Macaca mulatta*) and humans [40–41]. The STM capacity (measured as number of remembered items), however, has been found to be higher in humans compared to rhesus macaques [40–44], though such differences might stem from encoding errors rather than differences in STM ability [42].

Even though STM has been examined in a few model species, and in one case was tested in a fairly large number of primates from a broad phylogenetic sampling [39], a systematic phylogenetic investigation of STM abilities among (nonhuman) primate species has never been conducted with the types of analyses that can now be brought to bear on such large data sets. Such a phylogenetic investigation will offer a way of interpreting apparent quantitative species differences in an evolutionary framework. Thus, a second aim of the *ManyPrimates* project pilot study was to present a large number of primate species with the delayed-response task (DRT), testing their STM using near-identical methods and applying new analytic techniques which could reveal phylogenetic factors that predict memory performance. Our task was modelled on the DRT used by Barth and Call [38], in which participants needed to remember the location of a food item hidden in one of three opaque cups over a 0- or 30-second retention interval. We decided to add a 15-second retention interval condition to reduce the risk of floor or ceiling effects (we did not add more retention intervals or hiding locations to constrain the individual’s workload to allow more sites to contribute to this project). We expected that performance would decline with increasing retention intervals. Moreover, we expected that performance would decline with increasing age of participants (standardised by the maximum recorded lifespan for each species). A decline in STM performance with age has been reported, for example, for humans (though declines tend to happen only late in life) [43]; and rhesus macaques [44].

In an exploratory analysis we performed phylogenetic statistics and estimated ancestral states. Given that we plan to increase our sample size in the future, these phylogenetic analyses are preliminary and should be taken primarily as proof of concept.

## Materials and methods

### Participating institutions

As the aim of the project was to establish a collaboration between different sites housing primates, the only conditions for participation were that the institution hosts at least one primate species and that data collection would be finished before August 1st, 2018. Ethical approval was obtained separately for each institution. For details on each site (including ethical approval), see S1 File.

### Subjects

Our final sample comprised 176 primates of various ages from 12 species housed at 11 different sites. S1 File lists the number of subjects for each species as well as the number of sites contributing data from this species. Additional subjects started participating but did not finish the experiments, so their data were not included (see S1 File).

### Materials

**Fig 1:**
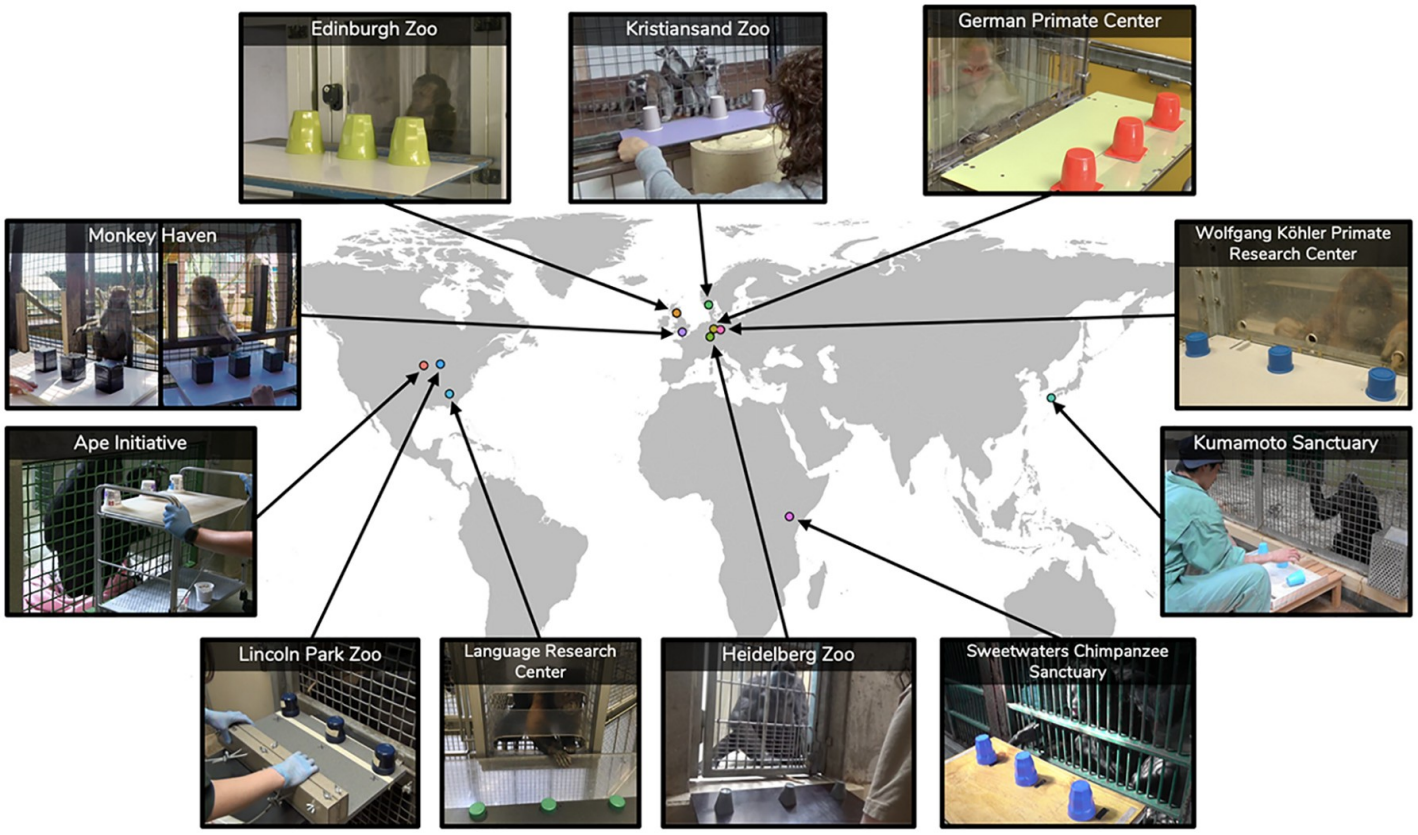
Test setup at the different sites. Clockwise, starting top left: Edinburgh Zoo, Kristiansand Zoo, German Primate Center, Wolfgang Köhler Primate Research Center, Kumamoto Sanctuary, Sweetwaters Chimpanzee Sanctuary, Heidelberg Zoo, Language Research Center at Georgia State University, Lincoln Park Zoo, Ape Cognition and Conservation Initiative, Monkey Haven (see S1 File for more details on sites).

The general setup comprised a rectangular board and three opaque cups (see Fig 1). Food items were used as rewards. For testing, the board was placed in front of the test subjects, outside their enclosure. The cups were evenly spaced on the board with at least 10 cm between them (center to center). The following aspects of the method varied between sites: board size, cup size and color, distance between cups (beyond 10 cm), food reward used, group vs. individual testing and experience with object choice tasks. While some variations were due to differences in body size between species (board size and distance between cups), others arose because of site-specific constraints (group vs. individual testing and task experience). S1 File lists site specific information for each of these variables.

### Procedure

Subjects were tested individually or in a group setting (see S1 File). For subjects tested in a group setting, the experimenter specified the focal individual ahead of time and tried to distract non-focal individuals during testing by giving them access to additional food or enrichment items. Test sessions were videotaped. Trials were scored live and confirmed from video. A coder unfamiliar with the purpose of the study additionally coded 20% of trials from video to obtain inter-rater reliability. The lowest measure of agreement between coders across sites was K = 0.90 (see S1 File for site specific information on inter-rater reliability).

At the beginning of a trial, the board was pulled back so that the subject could not reach it. The experimenter (E) stood or sat down behind the board and placed the three cups next to each other with the opening facing the subject. Next, E showed the subject a food item and placed it in front of one of the cups. Then E put the cups down one by one, thereby hiding the food item, always starting with the cup on the left from E’s perspective. Depending on the condition, E either waited for 0, 15, or 30 seconds before pushing the board towards the subject. While pushing the board forwards, E looked down, so as not to cue the subject. The subject made a choice by either pointing to or touching one of the cups. If the subject pointed to or touched more than one cup simultaneously, the trial was not counted and repeated. The trial was also repeated if the subject did not make a choice within 60 s. After the subject chose a cup, E pulled the board back and lifted the indicated cup. If the cup revealed the food item, the subject received it as a reward. If the cup did not cover the food item, the subject received no reward and the same food item was used again in the next trial. Finally, E put the cups back into the starting position for the next trial, which began immediately.

The hiding location was randomized across trials with the following constraints: The same location occurred no more than two times in a row and each hiding location occurred an equal number of times per condition. Trials were grouped in blocks, with each block comprising three trials of the same condition (0, 15, and 30 seconds). Blocks were grouped in sessions and the order of conditions was randomized within sessions. The number of trials per session varied between sites, species and individuals, with the constraint that there were at least nine trials (three blocks, one from each condition) per test day. This constraint was not always met. The total number of trials (and sessions) also varied between sites, species and individuals. We initially planned to present at least 18 trials and a maximum of 36 trials per subject. However, some individuals in the final sample contributed fewer than that (min. 6 trials).

Subjects without any experience in object choice tasks received additional training to ensure reliable choice behaviours. They had to reliably point to, or reach for, visible food items placed on the board before the start of the experiments. The duration of training varied between subjects. The number of subjects without task experience can be found in S1 File. Sessions were terminated if a subject did not make a choice in three consecutive trials. A new session was started the next testing day. Subjects were further excluded from the study if three consecutive sessions had to be terminated because the subject did not make a choice.

We coded which cup the subject touched or pointed to after the board had been pushed forward. If the indicated cup covered the food, the response was coded as correct. We also coded the location of the indicated cup (left, middle, right—from E’s perspective). In addition to this primary response, we coded a secondary choice, i.e. whether the subject indicated a second cup before the content of the cups had been revealed. All confirmatory analysis reported above are based on first response only.

## Results

Design, procedure and confirmatory analysis were pre-registered prior to data collection at the Open Science Framework (https://osf.io/v5je6/). The data and analysis code can be accessed at https://github.com/ManyPrimates/mp_pilot.

### Confirmatory analysis

**Fig 2:**
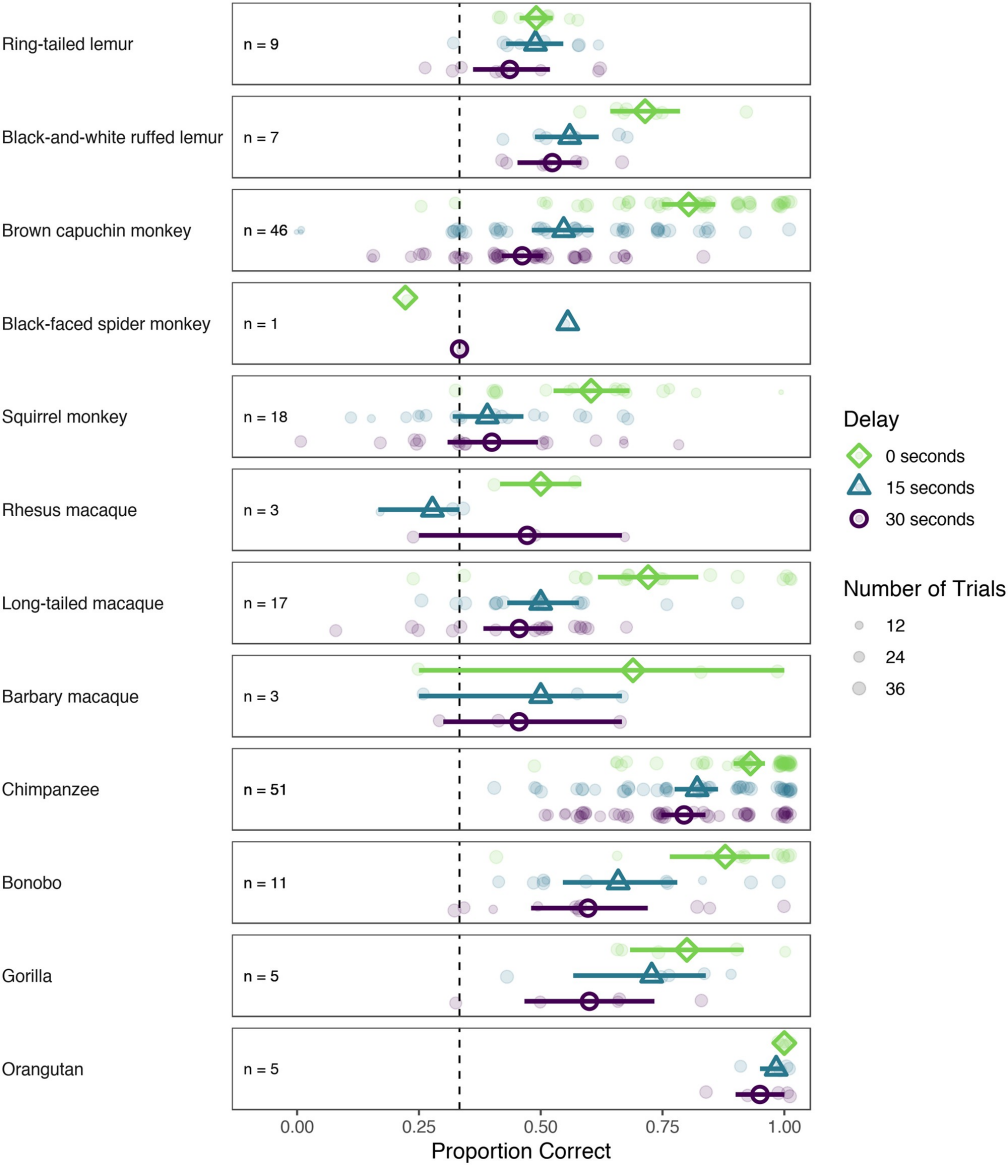
Proportion correct choice by species and condition. Open shapes denote the group mean per condition. Error bars are 95% confidence intervals based on a nonparametric bootstrap. Small, transparent dots represent aggregated data (mean proportion correct) for each individual. Size of these dots is proportional to the number of trials that individuals completed. Dashed line indicates performance expected by chance.

All analyses were run in R (version 3.5.1; [45]). As a first step, we aggregated the data across trials for each individual and condition within species and compared the proportion of correct choices to a level expected by chance (0.33). We excluded all species with three or fewer individuals (Barbary macaque, rhesus macaque, spider monkey) from this analysis. This analysis roughly indicates whether subjects from a given species were able to remember the hiding location for a given length of delay. We used one-sample *t*-tests for statistical comparisons. S1 File and Fig 2 summarize performance by species compared to chance.

For the main analysis, we fitted a generalized linear mixed-effects model (GLMM) to the trial-by-trial data using the function *glmer* from the package *lme4* [46]. The main goal of the confirmatory analysis was to investigate how the length of the delay influenced subjects’ ability to remember the correct location. For this predictor variable, we treated each length of delay as a discrete factor level (0, 15 and 30 seconds). To compute pairwise contrasts between levels of delay, we used the *emmeans* function and package [47]. We hypothesized that the effect of delay would be moderated by age and therefore included an interaction term between age and delay in the model. To make age comparable across species, we divided each subject’s age by the maximum recorded lifespan of that species (see http://genomics.senescence.info/species/) to yield a measure of which proportion of the maximum lifespan a subject had already lived. We then normalized these proportions across species. To evaluate the interaction term as a whole, we used likelihood ratio tests (LRT) using single-term deletions, as implemented in the function *drop1* from the package *stats*.

Additionally, we included the following fixed effects in the model: Task experience reflected whether or not the subject had experience with object choice tasks. Cup distance and board size (both in cm) captured site-specific variations in the physical setup. Trial number (continuous across sessions) was included to assess learning effects over time. Predictors for age, cup distance, board size and trial number were standardized. Random effect structure was maximal and is presented in the model formula below. We computed bootstrap confidence intervals with 1,000 replicates. We had pre-registered the following model:

correct ~ delay * age +

 task_experience + cup_distance + board_size + trial +

 (1 + delay + trial | site/subject/block/hiding_location) +

 (1 + task_experience + cup_distance + board_size + trial + delay | species)

The maximal converging model had the following structure:

correct ~ delay * age +

 task_experience + cup_distance + board_size + trial +

 (1 + delay + trial | site/subject/block) +

 (1 + task_experience + cup_distance + board_size + trial + delay | species).

Next, we removed random effects with near-zero variance. Details of the pruning procedure can be found in the analysis script. The two models do not differ in the kind of inference they license about delay, age, cup distance and board size. The model presented in the main text yielded a lower AIC value, suggesting a better out-of-sample fit. The final model had the following structur:

correct ~ delay * age +

 task_experience + cup_distance + board_size + trial +

 (1 + delay | site/subject) +

 (1 + delay | species)

**Fig 3:**
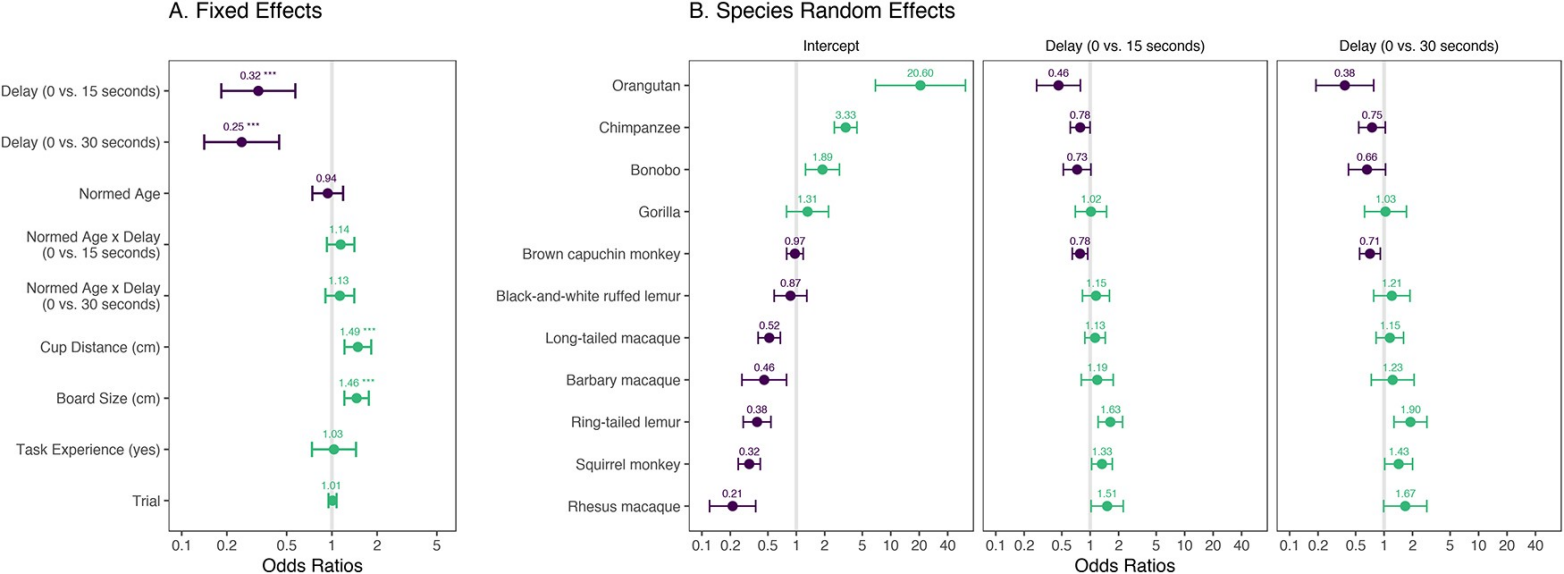
Forest plots for GLMMs predicting mean proportion of correct choices. A. Odds ratios for fixed effects in the final model. B. Odds ratios for species random effects in the model with a reduced random-effects structure. Odds ratios are plotted on a log scale.

Fig 3A shows odds ratios (OR) for the fixed effects based on the model. The model estimates confirm the hypothesized retention effect. Across species, performance was better in trials with shorter delays (0 vs. 15 seconds: *β* = -1.13, 95% CI [-1.65, -0.56], *p* < .001, OR = 0.32; 15 vs. 30 seconds: *β* = -0.25, 95% CI [-0.43, -0.08], *p* = .011, OR = 0.77).

Contrary to our hypothesis, we found no interaction between age and delay (LRT: χ^2^(2) = 1.57, *p* = .456). The model also showed positive effects of cup distance (*β* = 0.40, 95% CI [0.19, 0.62], *p* < .001, OR = 1.49) and board size (*β* = 0.38, 95% CI [0.20, 0.59], *p* < .001, OR = 1.46) on performance. See S1 File for a more detailed discussion of these results.

We evaluated species differences by extracting and plotting random effects from a model that included the same fixed effects as the final model but only species as a random effect with a random slope for delay (Fig 3B). The left facet shows how each species’ intercept differs from the mean intercept. This roughly corresponds to variation across species in the 0-second delay condition while taking into account all other model predictors. Higher odds ratios indicate better performance. The middle and right facets show the extent to which, according to the model, species differed in their performance in the delay conditions. In this case, higher odds ratios suggest that a species showed a larger performance difference between the 0- and 15-second delay (or between the 0- and 30-second delay, respectively) compared to the mean estimate for that difference. In sum, the confirmatory analysis provides evidence for the hypothesized retention effect and showed substantial variation in performance across species and conditions. The latter is an important prerequisite to address phylogenetic questions, to which we now turn.

### Exploratory analyses

#### Phylogenetic analyses

There were two main goals of our phylogenetic analyses. First, we estimated the strength of the phylogenetic signal in our data, that is, to what extent is short-term memory ability directed by the structure of primate phylogeny? Second, we made inferences about how short-term memory evolved over time. We used the package *phytools* for these analyses [48].

#### Phylogenetic signal: λ

To address the first question, we estimated the strength of the phylogenetic signal with a single value known as “lambda” or λ. Put simply, λ reflects whether similarities in different traits across species are as similar as one would expect, given a shared evolutionary history. λ ranges (in most cases) between 0 and 1, with 0 indicating no signal and 1 indicating a very strong signal. The statistical significance of λ can also be evaluated using likelihood ratio tests. For more on λ and phylogenetic signal, see [18], or for a less technical example, see [49].

For our purposes, λ could be estimated two different ways. The first examines performance means in isolation. In the second approach, λ can be estimated as part of a phylogenetic t-test. This type of t-test is essentially a one-sample t-test that recognizes that species might be more similar to each other than one might otherwise expect, due to shared evolutionary lineage. The additional information that is considered in this type of t-test is the standard deviation and reference (i.e. chance performance) level. One can think of the first type of λ as analogous to a base λ and the second type is an updated λ, i.e. from a more complex model.

We estimated STM ability across all trials in the 15- and 30-second delay conditions and tested this against chance performance (0.33) in the eleven species for which data from more than one individual were available. We excluded the 0-second delay trials because the short-term memory component was minimal, if present at all. Base λ of the memory abilities was 0.79, though this base model value was not itself significant (*p* = 0.267). We then evaluated the updated λ in a t-test (*t* = 3.35, *df* = 8, *p* = 0.010), which suggested that across primate phylogeny, primates can generally perform this task above chance. This t-test incorporated all our information on short-term memory abilities and yielded an updated λ = 0.999 (*p* = 0.010), indicating that there was substantial and significant phylogenetic signal in our data. In other words, species that share more evolutionary history with one another will tend to perform more similarly on short-term memory tasks.

#### Reconstruction of the ancestral state

**Fig 4:**
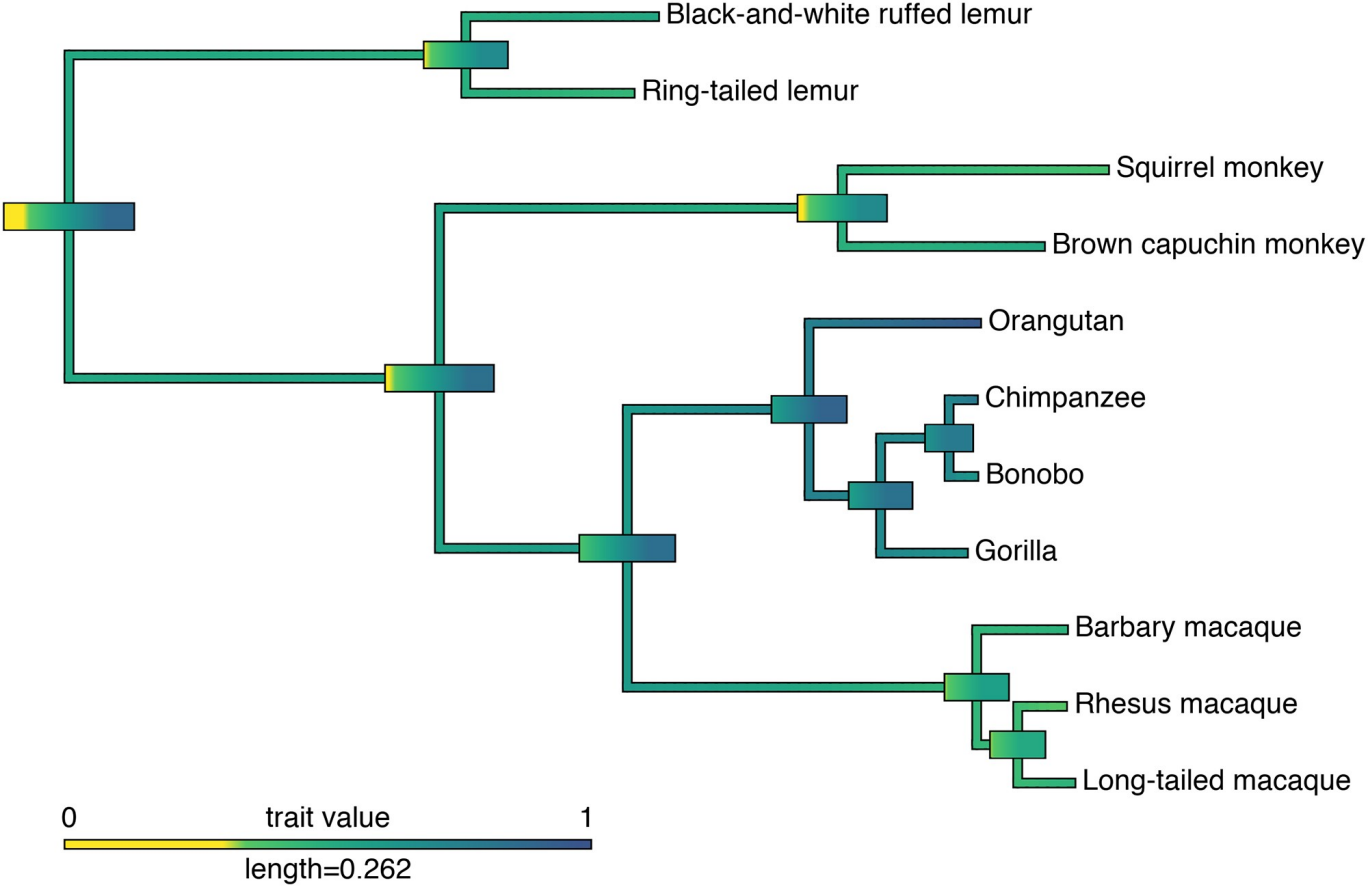
Ancestral state reconstruction of primates’ short-term memory abilities for the 15- and 30-second delay conditions. Colors are representative of the confidence intervals of abilities, for this trait, accuracy ranging from 0 to 1. Large bars represent the confidence intervals of proposed common ancestors. Any yellow in the confidence interval indicates below chance performance. Length of legend indicates scale for the branch lengths (proportional to the number of nucleotide substitutions per site). Phylogram was obtained using the 10kTrees primate dataset [50]. *Pan t*. *verus* and *Pongo abelii* were arbitrarily chosen as the representative species or subspecies of chimpanzees and orangutans. This was because detailed species and subspecies information was not available at this point in the study; the results were not affected by these decisions.

Phylogenetic data allow researchers to model traits of hypothetical common ancestors [48]. The goal of so-called ancestral state reconstruction is to estimate phenotypic traits at internal branching points, or nodes, in a phylogenetic tree. In the case of primate cognition and short-term memory capacity, we were interested in how recently stronger memory aptitudes evolved and where along the tree these adaptations occurred (Fig 4).

Lemurs, New World monkeys, Old World monkeys, and apes are all capable of exceeding chance levels of performance in the 15- and 30-second delay conditions of the short-term memory task. These clades all performed similarly. The strongest short-term performance is seen in great apes, who, as a group, perform much better than the other clades. In the theoretical common ancestors of these clades, only the ape and the broader ape/Old World monkey ancestors were estimated to perform above chance.

This suggests that while short-term memory ability had been developing in Old World monkeys, the strong working memory abilities of humans and other apes evolved after the divergence of apes from Old World monkeys. However, we represented only a small minority of primate species in our analyses, resulting in a small sample size with many missing data points along the branches of the primate lineage. The analysis presented here is therefore more an illustration of what type of questions can be addressed in phylogenetic analysis rather than an attempt to answer them. Additional data from more primate species will be necessary to make valid inferences about the evolution of short-term memory.

## Discussion

### Summary of study and findings

This study reports short-term memory capacities in 12 non-human primate species, based on over 170 test subjects that were housed in 11 different sites located in Africa, North America, Asia and Europe. Thereby, the study provides the most comprehensive and comparable dataset of primate short-term memory to date, with data for apes, monkeys, and prosimians. We aimed to replicate findings from several small-scale studies and expand upon them by testing yet untested species in addition to already-studied ones. All subjects were tested in a delayed-response task, following the same methods, in which individuals could gain food rewards if they remembered the position of a hidden reward for 0, 15, and 30 seconds. As predicted, the shorter the delay, the better the individuals performed. This diverse sample size was achieved by the establishment of an international collaborative framework, the *ManyPrimates* project. In addition to providing novel insights into primate short-term memory, this study highlights that such a large-scale collaboration in the field of primate cognition is feasible and valid.

Short-term memory abilities appeared to be closely linked to the evolutionary relatedness of the different species that participated in these tests. In attempting to reconstruct the ancestral states of short-term memory abilities across the primate lineage, we found that following the divergence of apes, short-term memory abilities appear to have developed considerably. This result, however, should be interpreted with caution. Even though our sample is large in terms of the number of individual participants, it is still small with respect to the number of species. Additionally, while our study did not reveal an effect of age on the primates’ performance in the task, this is possibly due to the large proportion of younger individuals in our sample. Future work with more species, including more diverse ages, is required to draw valid conclusions about the evolution and development of short-term memory.

### An infrastructure for collaboration

The main goal of this pilot study was to establish an infrastructure on which future studies can be built. We began by examining an aspect of cognition that has, historically, been among the first capacities investigated in any new species to be studied: memory [36, 37, 51–53]. While some of the species that we included have been studied in similar tests in isolation, our efforts bring together one of the largest samples ever studied in primate cognition, with all subjects tested following very similar protocols. We managed to conceive, plan and conduct this study in less than 10 months. We did so without any dedicated external funding specific for this project. This shows that large-scale, international studies can be organized and conducted in an efficient way with nonhuman species at sometimes highly variable facilities. The efforts of this team also indicate that such collaboration can be productive and collegial, provided there is a defined purpose, consensus on research aims, and a precise protocol for all team members. Overall, the logistical success of this project shows that the infrastructure we developed is suitable to host future studies. In the following we provide some more details on the nature of this collaboration.

The first task for a multisite collaboration project is the establishment of an infrastructure that enables researchers to communicate and coordinate. Collaboratively discussing ideas and agreeing on topics and protocols requires ways of reaching the people involved in the various aspects of the project. In the case of *ManyPrimates*, we use a mailing list as a central platform for communication. This list is mainly used to announce new developments and summarize discussions. For in depth discussions, we use a group messaging tool (Slack).

Another challenge is making the project known to people in the field in order to get them involved. So far, we have used our website (https://manyprimates.github.io/), social media (Twitter: @ManyPrimates), and conference presentations [54,55] for recruiting and outreach activities. The most effective method for getting new people involved so far has been word of mouth. This article presents yet another attempt to reach a wider audience.

The effective distribution of work among members of the group requires tools for collaborative file management. We use cloud-based word processors for writing guidelines, procedures, coding instructions, pre-registrations, and manuscripts. For storing data and analysis scripts, we use a web-based hosting service (https://github.com/ManyPrimates). These tools allow multiple people to access and concurrently edit files remotely.

### Challenges of multisite collaborations

Testing a wide variety of species with varying physical, social, and psychological characteristics poses some presumably unique challenges to a collaborative research project. In order to include a wide variety of species in the study, the design and setup needs to be adjusted to species and site-specific constraints. *ManyPrimates* studies that seek to replicate earlier findings are therefore always conceptual rather than direct replications. Furthermore, variation in design and setup might cause other problems. In the case of the pilot study presented here, the most obvious problem was that aspects of the physical layout were correlated with species (size) and site. This resulted in a statistical effect of cup distance and board size on performance. Even though we have good reason to believe that cup distance and board size have no direct effect on performance, we cannot rule this possibility out at this point.

Subjects also varied in terms of their research experience. Although task experience did not have a statistical effect in the present study, it will be important to document this in future studies and for other cognitive capacities. For example, testing experience in certain perceptual discrimination tasks would likely impact later performance in similar tasks. Similarly, experience with tool-using tasks may impact later tool-use performance in new problems. Furthermore, we used a very narrow definition of task experience and only documented whether subjects had experience with tasks that involved choosing between alternatives using pointing. This should be broadened in the studies to come. In addition, task experience might have motivational consequences: Participation is usually rewarded with food, and individuals who regularly participate in studies might be more motivated to solve a problem in anticipation of a reward. Continuing to make the experience factor a central focus of our efforts will allow us to estimate the generalizability of results found in a particular sample to the broader population.

Another challenge comes with differences in testing arrangements across sites. At some sites, individuals cannot be separated, and therefore testing happened in a group setting. This arrangement might influence performance due to a noisy, distracting, or competitive testing environment. The more severe consequence, however, is that, because some individuals can monopolize the task, others end up not contributing data at all. This makes it impossible to investigate whether individuals who do and do not contribute data differ in characteristics that are relevant to the task. In the future, we will aim to at least document this selective attrition rate for groups in which only some individuals participate [53].

Our strategy regarding the aforementioned difficulties has been to document them. This approach will enable us, whenever possible, to test their influence on the dependent measure and, in the remaining cases, specify the limitations of the results. In the future we will also try to induce variation in potential confounds within and between species, to test whether they may influence task performance. This will help to address some of the shortcomings that come with including many different species in one research design, yet they will not address all limitations. A careful interpretation of results is therefore always warranted and alternative interpretations should be addressed empirically.

### Future of *ManyPrimates*

We want to continue the data collection for short-term memory. Even though the present results are impressive when compared to past research on this topic, the sample size and number of species are still too small to address phylogenetic questions in a rigorous way. Thus, we hope to expand our data collection efforts with this protocol, testing more subjects and species. At the same time, we are also discussing topics and settings for future *ManyPrimates* studies. At the moment, the majority of people involved in the project work with captive primates. Our goal is to get more field primatologists involved to exchange ideas about ways of testing wild and captive primates in a comparable study design. Furthermore, we are open for collaborations with other projects (e.g. *ManyLabs*, *ManyBabies*) to also include humans participants in our studies. Eventually, we hope that the *ManyPrimates* infrastructure will be used to simultaneously plan and coordinate multiple studies on a wide range of topics and with a diverse range of species in different settings.

*ManyPrimates* provides many different opportunities for contribution. Central and crucial contributions are, of course, enabling access to primates and collecting data. These components have a profound influence on the scope and therefore the success of the project. However, there are many ways to contribute beyond direct involvement in data collection. Some examples are facilitating and coordinating discussions of study topics, writing protocols, coding schemes and coding instructions, coding data, curating and analysing data as well as preparing manuscripts for publication. These aspects provide a unique opportunity for researchers, often in an earlier career stage, without direct access to primates to make a contribution. Our authorship guidelines (see: https://manyprimates.github.io/authorship/) specify the different ways in which potential contributors can gain authorship on project publications.

Large-scale collaboration also allows institutions with only a small number of primates from each species to make an important contribution. Similarly, many species (e.g., gibbons) are notoriously understudied because very few institutions alone have access to a large enough sample. When combined across sites, these numbers become sufficiently large. This is also an opportunity to draw attention to primates that are less studied, or have not been studied at all (e.g. critically endangered species). Furthermore, collaborative endeavors like the one presented here are also more likely to attract funding, which can be used to support data collection at sites that otherwise could not contribute.

Finally, *ManyPrimates* opens up new conservation possibilities; phylogenetic approaches can help conserve rare species in the wild by allowing inferences about their behaviour to be made from other closely related species [56]. This is particularly important given the growing number of endangered primate species [57]. Participation in cognitive testing may also improve subjects’ welfare (see e.g. [58–62]), depending on the complexity of the task [63] and the manner in which the primates are tested [64]. Future projects could collect the primary research data and also include evaluations on the efficacy of the research activity to be enriching to test subjects [65–67]. Taken together, we encourage everyone interested in primate cognition research to get in touch and get involved. It is our sincere hope that future studies will greatly surpass the one presented here in scope and size.

### Conclusion

We present the *ManyPrimates* project, a large-scale collaboration project, initiated to address an important problem in comparative psychology: the difficulty of collecting data necessary to adequately reconstruct the evolutionary history of primate behaviour and cognition.

Our pilot study generated one of the most comprehensive and diverse datasets of primate short-term memory to date, demonstrating that the infrastructure we implemented is effective. There are, however, important limitations. The results of the phylogenetic analysis are preliminary because of the relatively low number of species we tested. However, using the infrastructure established here, we hope to recruit larger numbers of individuals and species in the future, enabling robust phylogenetic analyses and valid inferences about the evolution of primate cognition.

The success of our pilot study demonstrates the feasibility of a large-scale collaboration between researchers and institutions across continents without dedicated external funding. As such, *Many Primates* provides an exciting opportunity to better understand the evolution of primate cognition and behaviour.

## Supporting information

S1 File Supplementary information. Includes information on site specific adjustments, testing arrangements and reliability coding, Sample size, number of sites and mean performance per species and condition in comparison to chance level, additional results for the effect of age, cup distance and board size on performance, comparisons across sites within species and detailed descriptions of each data collection site including ethical approval.
(PDF)
