# Correction: Establishing an infrastructure for collaboration in primate cognition research

**DOI:** 10.1371/journal.pone.0225236

**Published:** 2019-11-07

**Authors:** The PLOS ONE staff

## Notice of republication

An incorrect version of the author byline was published in error. This article was republished on October 28, 2019 to correct for this error. Please download this article again to view the correct version.
