# Supplementary material for: Establishing an infrastructure for collaboration in primate cognition research
Source: PLoS One. 2019 Oct 24;14(10):e0223675. doi: 10.1371/journal.pone.0223675 (PMC6812783; doi:10.1371/journal.pone.0223675)
Supplement: S1 File — Includes information on site specific adjustments, testing arrangements and reliability coding, Sample size, number of sites and mean performance per species and condition in comparison to chance level, additional results for the effect of age, cup distance and board size on performance, comparisons across sites within species and detailed descriptions of each data collection site including ethical approval. (PDF) [file pone.0223675.s001.pdf]

Establishing an infrastructure for collaboration in primate cognition research

S1 supplementary information

Site specific adjustments, testing arrangements and reliability coding

Table S1. Site specific adjustments, testing arrangements and reliability coding

Site	Species	Board size (l x h) in cm	Cup distance in cm	Test setting	Subjects without task experience	Drop-outs	K[1]
Kristiansand Zoo	Ring-tailed lemurs and black-faced spider monkeys	74.5 x 27.5	10	Group	3	3	1.00
Edinburgh Zoo	Brown capuchin monkeys	60 x 30	10	Individual	0	0	0.97
	Squirrel monkeys	60 x 30	10	Individual	0	2	1.00
	Chimpanzees	64 x 14	18.5	Group	0	0	0.98[2]
Ape Cognition & Conservation Initiative	Bonobos	51 x 38	20	Individual	0	0	0.96
Monkey Haven, Isle of Wight	Rhesus and barbary macaques	55 x 45	10	Individual	0	1	1.00
German Primate Center	Long-tailed macaques	53 x 28 & 60 x 40	10	Individual	0	5	0.99
	Ring-tailed lemurs & black-and white ruffed lemurs	63 x 30	10	Individual	0	1 ring-tailed lemur	0.97
Lincoln Park Zoo	Gorillas	61.6 x 35.6	19	Individual	3	0	0.90
Kumamoto Sanctuary	Chimpanzees & bonobos	60 x 30	15	Individual/ Group	12	0	1.00
Wolfgang Köhler Primate Research Center	Chimpanzees	78 x 38	29	Individual	0	1	1.00
	Orangutans	78 x 38	29	Individual	0	1	1.00
Sweetwaters Chimpanzee Sanctuary	Chimpanzees	80 x 30	20	Individual	1	0	0.97
Heidelberg Zoo	Gorillas	75 x 40	20	Individual/ Group	3	1	1.00
Language Research Center	Brown capuchin monkeys	56 x 20	10	Individual	0	1	0.97

Note. [1] K = Cohen's Kappa; based on coding of at least 20% of trials by a coder unfamiliar with the purpose of the study. [2] Second coder was not unfamiliar with purpose of study.

Supplementary results

Performance compared to chance

Table S2. Sample size, number of sites and mean performance per species and condition in comparison to chance level (0.33).

Species	*N*	Sites	Delay (in seconds)	*M*	*d*	*t*	*p*	
Ring-tailed lemur	9	2	0	0.49	2.75	8.25	< .001	***
			15	0.49	1.63	4.89	.001	**
			30	0.44	0.79	2.37	.046	*
Black-and-white ruffed lemur	7	1	0	0.71	3.59	9.51	< .001	***
			15	0.56	2.44	6.45	.001	**
			30	0.52	2.05	5.43	.002	**
Brown capuchin monkey	46	2	0	0.80	2.46	16.69	< .001	***
			15	0.55	0.92	6.25	< .001	***
			30	0.46	0.87	5.91	< .001	***
Black-faced spider monkey	1	1	0	0.22	-	-	-	
			15	0.56	-	-	-	
			30	0.33	-	-	-	
Squirrel monkey	18	1	0	0.60	1.57	6.67	< .001	***
			15	0.39	0.34	1.44	.168	
			30	0.40	0.33	1.35	.197	
Rhesus macaque	3	1	0	0.50	-	-	-	
			15	0.28	-	-	-	
			30	0.47	-	-	-	
Long-tailed macaque	17	1	0	0.72	1.73	7.12	< .001	***
			15	0.50	1.05	4.33	.001	**
			30	0.46	0.78	3.23	.005	**
Barbary macaque	3	1	0	0.69	-	-	-	
			15	0.50	-	-	-	
			30	0.46	-	-	-	
Chimpanzee	51	4	0	0.93	5.06	36.17	< .001	***
			15	0.82	2.91	20.80	< .001	***
			30	0.79	2.77	19.76	< .001	***
Bonobo	11	2	0	0.88	2.97	9.83	< .001	***
			15	0.66	1.64	5.43	< .001	***
			30	0.60	1.23	4.07	.002	**

Gorilla	5	2	0	0.80	3.08	6.89	.002	**
			15	0.73	2.15	4.80	.009	**
			30	0.60	1.40	3.14	.035	*
Orangutan	5	1	0	1.00	-	-	-	
			15	0.98	17.44	39.00	< .001	***
			30	0.95	8.27	18.50	< .001	***
Total	176	11						

Note. For the spider monkey, rhesus macaques, and barbary macaques, we did not compute *t*-tests due to the small sample sizes. For orangutans in the 0 seconds delay condition, we could not compute a *t*-test because the data were constant; all orangutans chose the correct option in every trial. * $p < .05$, ** $p < .01$, *** $p < .001$

Effect of age on performance

Contrary to our hypothesis, we found no interaction between age and delay (LRT: $\chi^2(2) = 1.57$, $p = .456$;). Fig S1 shows the relation between age and performance for the different lengths of delay. This plot also shows that there were few very old individuals in our sample and that old individuals also tended to be apes, who had a higher performance on average. Thus, a more balanced sample might be necessary to examine the relation between age and delay in a comprehensive way.

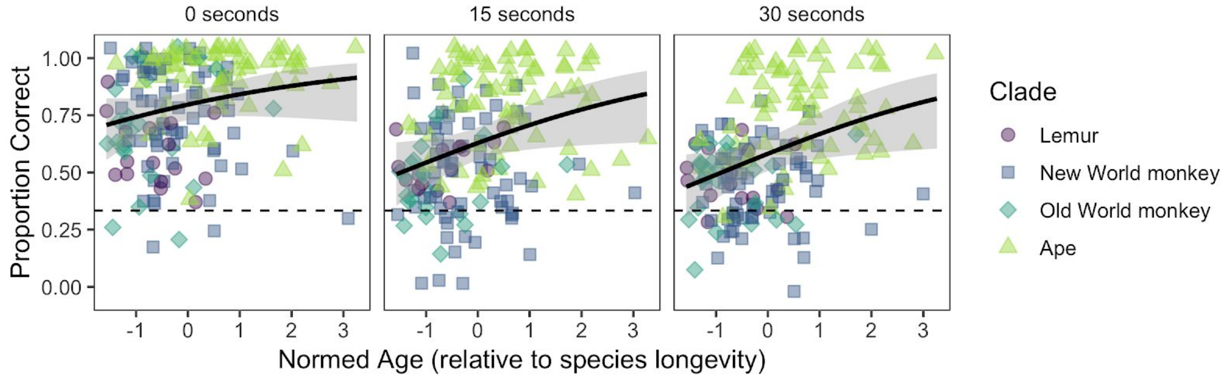

Fig S1. Proportion correct choice as a function of age (normed by species longevity), delay and clade. Dashed line indicates performance expected by chance. Black line and shaded area indicate regression line and 95% confidence interval.

Effects of cup distance and board size on performance

The model also showed positive effects of cup distance (*β* = 0.40, 95% CI [0.19, 0.62], $p < .001$, OR = 1.49) and board size (*β* = 0.38, 95% CI [0.20, 0.59], $p < .001$, OR = 1.46) on performance. These effects might seem surprising, however, a closer look at the data showed that cup distance and board size co-varied with species and site (see Fig S2). For example, only apes had a cup distance larger than 11 cm and all orangutans (who had the highest performance overall) came from the site with the largest cup distance. Future data collection should vary both aspects more evenly across species and sites.

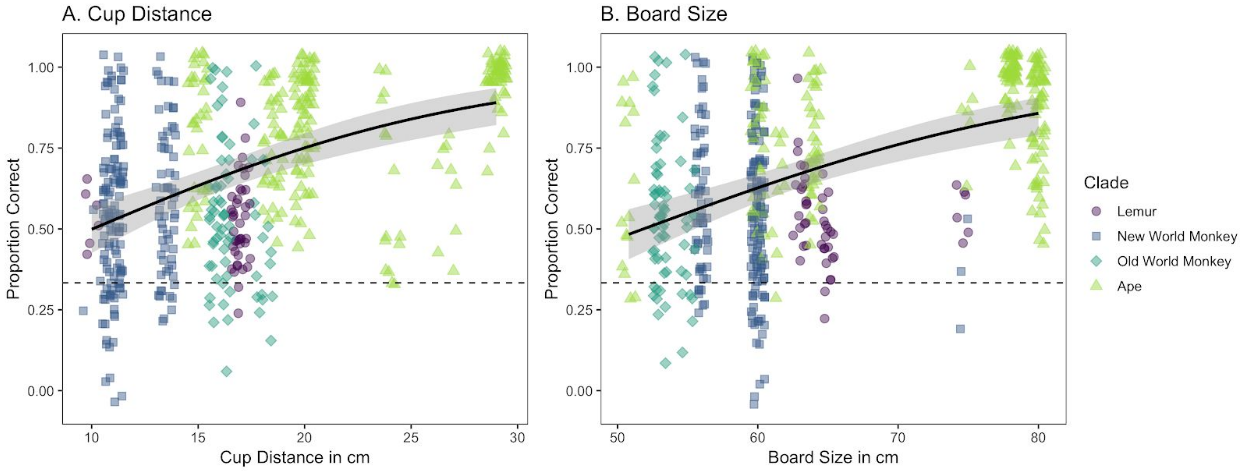

Fig S2. Proportion of correct choices by cup distance (A) and board size (B) for each clade. Dashed line indicates performance expected by chance. Black line and shaded area indicate regression line and 95% confidence interval.

Comparing performance across sites

For many of the species, there was not a sufficient number of individuals from each site to consider site-specific performance differences; notable exceptions were the data collected for chimpanzees and capuchins. We collected data from chimpanzees at five different sites, but performance was remarkably similar across sites (Fig S3) except at the Wolfgang Köhler Primate

Research Center, where performance was the highest. Similarly, among capuchin monkeys, for which we had data from more than 20 individuals at each of two sites, performance between sites was remarkably similar (Fig S3).

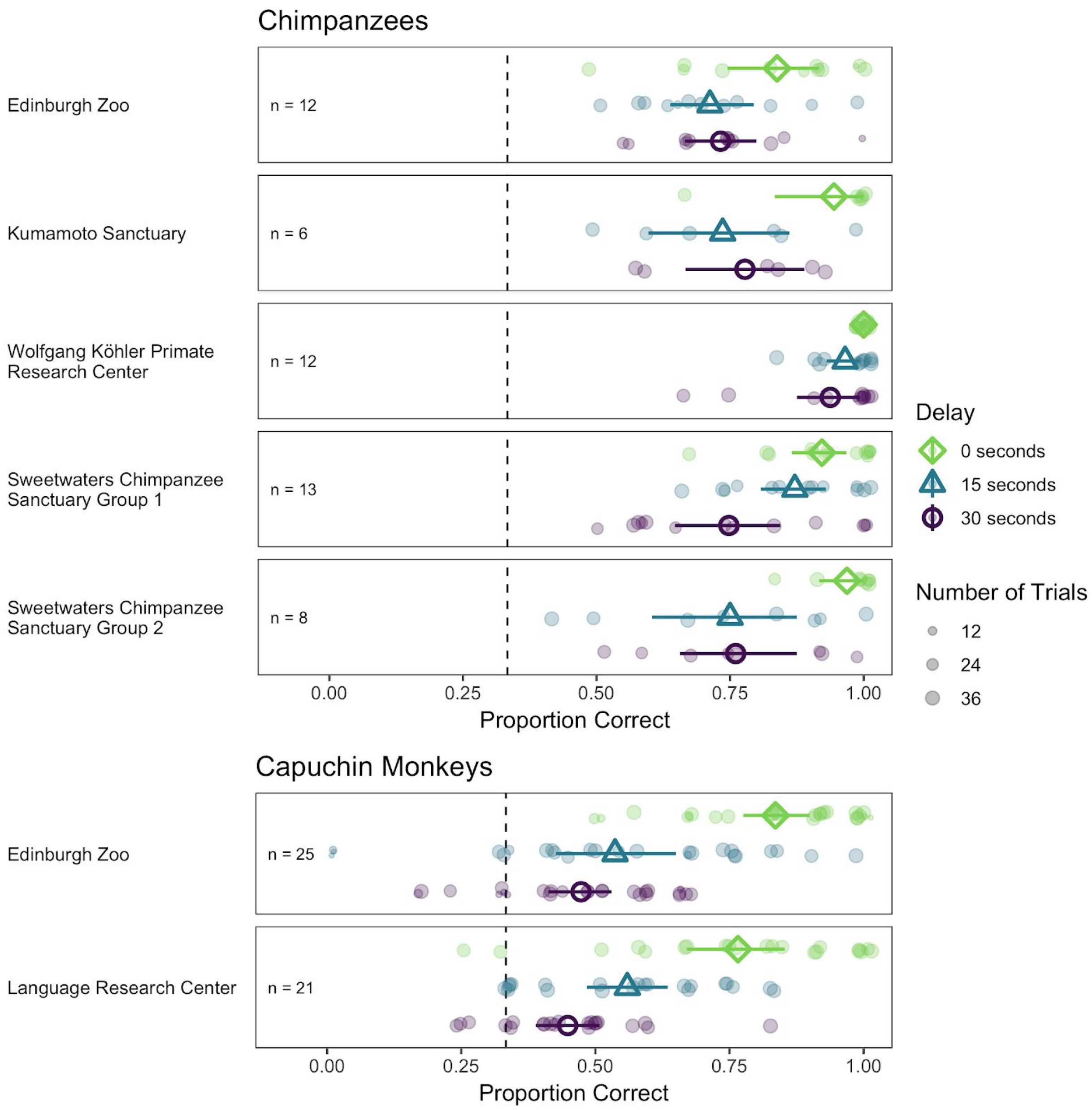

Fig S3. Comparison of performance across different sites, for chimpanzees and capuchin monkeys. Open shapes denote the group mean per condition. Error bars are 95% confidence intervals based on a nonparametric bootstrap. Small, transparent dots represent aggregated data (mean proportion correct) for each individual. Size of these dots is proportional to the number of trials that individuals completed. Dashed line indicates performance expected by chance.

Data collection sites (including details on ethical approval)

Kumamoto Sanctuary

Background

Kumamoto Sanctuary (KS) is the first and only sanctuary for chimpanzees and bonobos in Japan.

Animals

54 chimpanzees and 6 bonobos live in KS. 6 chimpanzees and 6 bonobos were included in this study.

Enclosures

Apes lived in an enriched environment with an outdoor compound (200~ 700 m^2) equipped with climbing structures and vegetation attached to indoor sleeping rooms (70~ 200 m^2). They lived in social groups consisting of 6-11 individuals.

Diet

Chimpanzees were given a variety of vegetables, fruits, nuts, and monkey chow three times a day, with additional enrichment items between the main meals. Water was available ad libitum. Neither food nor water was deprived for the purpose of experiments.

Ethical approval

Wildlife Research Center, Kyoto University

Research permission

Animal husbandry complied with international standards (the Weatherall report "The use of non-human primates in research") and institutional guidelines (Wildlife Research Center "Guide for the Animal Research Ethics"). The experimental protocols were approved by the Ethics Committee of the Wildlife Research Center, Kyoto University (WRC-2018-KS008A).

Research training

Experimenters were trained to safely interact with apes and not to give subtle behavioral cues (e.g. gaze to the correct location) during the test for a minimum of 3 months.

Research participation

All apes were tested in indoor sleeping rooms for each species. Upon testing, each individual ape was invited from the outdoor compound to the indoor sleeping room. Then, the door between the

sleeping room and the outdoor compound was shut down to prevent other apes from coming in. All apes were willing to participate in the tests, and did not show any strong stress behaviors (e.g. stress defecation) in this study. When chimpanzees stop participating in the experiments and/or show such strong stress behaviors (e.g. upon hearing conspecific fights outside), we let them out by opening the door.

Other

No breeding program is adopted in the sanctuary.

Language Research Center, Georgia State University

Background

The Language Research Center (LRC) is an interdisciplinary research unit of the College of Arts and Sciences at Georgia State University. Although it was founded in 1981, its history begins a decade earlier in the ape-language research of founding Director Duane M. Rumbaugh and his collaborators. Historically, the LRC has housed and tested the cognition of bonobos, chimpanzees, orangutans, rhesus macaques, capuchin monkeys, and human children. At present, the LRC houses capuchin monkeys and rhesus monkeys.

Animals

The LRC contributed data from 21 capuchin monkeys.

Enclosures

The capuchin monkeys are housed in the CapLab, a separate facility with indoor (605 square feet) and outdoor (~1,450 square feet) areas and individual test cages for each animal, as well as group testing cages for observations of two or more animals. Additional (human-only) areas in this facility include areas for storage of cleaning supplies and personal protective equipment for staff, a kitchen area for storing and preparing food for the monkeys, and office space for record-keeping and experimental apparatus storage.

Diet

All animals are maintained on a veterinarian-approved daily diet that is supplemented with any food rewards used during cognitive testing. No food or water restriction is ever used with these monkeys.

Ethical approval

This research was approved by the IACUC of Georgia State University. Georgia State University is accredited by the Association for Assessment and Accreditation of Laboratory Animal Care International.

Research permission

Research with animals at Georgia State University is approved by the IACUC committee under protocol A19042.

Research training

All individuals are trained by the care staff and research staff at the Language Research Center under standard operating procedures approved by the IACUC.

Research participation

All monkeys participate at their own choosing, voluntarily entering tests areas when offered the opportunity to engage in cognitive testing. No participation is ever coerced.

Other

Monkeys are monitored daily by research and care staff for psychological and physical wellbeing.

Lincoln Park Zoo

Background

Lincoln Park Zoo is located in Chicago, USA. The zoo is a leader in local and global conservation, animal care and welfare, learning, and science. A historic Chicago landmark founded in 1868, the not-for-profit Lincoln Park Zoo, is a privately-managed, member-supported organization and is free and open 365 days a year. Currently, the zoo is home to around 200 animal species, including the gorillas who participated in this study.

All data collected at Lincoln Park Zoo for this study were collected by members of the zoo's Lester E. Fisher Center for the Study and Conservation of Apes. The Center's mission is to conduct multidisciplinary research that will 1) advance the knowledge of primate behavior, biology, and cognition, 2) evaluate and enhance the welfare of primates in zoos and sanctuaries, 3) conserve and protect wild primate populations and their ecosystems, and 4) provide unique training and educational opportunities for the next generation of primatologists.

Animals

Three gorillas from Lincoln Park Zoo were subjects in this study. The three were males: Azizi (age 14), Amare (age 12) and Mosi (age 11). They were housed together in an all-male group of four males in the Regenstein Center for African Apes at Lincoln Park Zoo, Chicago, USA. Also housed in the Regenstein Center of African Apes at the time of testing was a mixed-sex gorilla group (1 male, 3 adult females, 3 juvenile females) and two mixed-sex chimpanzee groups (one comprised of two adult males and four females, and the second comprised of two adult males and three females).

Enclosures

The three male gorillas that participated in this study lived in a complex exhibit and were provided with novel enrichment on a daily basis. A detailed description of this enclosure can be found in Ross et al. (2011). The indoor space featured a deep mulch floor, climbing structures, hammocks and visual barriers. The outdoor space had a grass floor, climbing structures, hammocks and visual barriers. They had access to their outdoor exhibit area whenever weather permitted. The total size of the indoor/outdoor exhibit was (1932 m^2). In addition to their large exhibit, where they spent most of their time (22-23 hours per day) there was also an off-exhibit "holding" area where they were moved to while animal care staff cleaned their exhibit. This is where testing took place (see below).

Diet

The gorillas were fed with a variety of fresh fruit and vegetables daily, in addition to primate chow. They were never food or water deprived. The food rewards that were used for this study (peanuts in the shell) were reviewed and approved by veterinary and nutrition staff prior to the start of the experiment.

Ethical approval

This study was approved by the Lincoln Park Zoo Research Committee (#2018-014), which is the governing body for all animal research at the institution. This research adhered to legal requirements in the United States of America and to the American Society of Primatologists' Principles for the Ethical Treatment of Nonhuman Primates.

This study (#2018-014) was approved by the Lincoln Park Zoo Research Committee (on June 1st 2018). The Lincoln Park Zoo Research Committee is chaired by the Zoo's Vice President of Conservation and Science, Dr. Lisa Faust, and is comprised of representatives from the zoo's full-time senior research staff (typically 10+ PhDs, 2-4 MSs), senior animal care staff (one veterinarian, the VP of Animal Care and Horticulture, and the General Curator), and at least one representative from the Learning department; the Vice President of Communications is ex officio to this committee.

Research permission

Participation in this study was approved by the Lincoln Park Zoo Research Committee.

Research training

The researchers are all employees of Lincoln Park Zoo and so have received extensive training working with these animals and run daily cognitive testing sessions with them for at least two years prior to the start of this study. Thus, not only are the researchers well trained, but the subjects were familiar with them.

Research participation

All participation was voluntary and, although not necessary, for this study, a session would be stopped if a subject showed any sign of distress. The gorillas were tested individually in their "holding" enclosures. The gorillas, who are part of an all-male group of four gorillas, are separated each morning by keeper staff as part of their typical husbandry routine. They are typically separated for no more than an hour and have continual visual, auditory and olfactory access with their group members while separated. All testing took place during this time.

Other

These gorillas are managed as part of the gorilla Species Survival Plan(R).

While the gorillas had extensive touchscreen research experience, they had not participated in many manual tasks of cognition. Therefore, before data collection could begin, we first had to train them to point to a food reward so that they could successfully indicated their selections during test. To do that, we placed a peanut in a shell (the same reward we used during testing) on the testing platform in view of, but out of reach of, a gorilla. Using positive reinforcement training and shaping techniques, we trained the gorillas to reach for the food rewards. Some individuals were more successful, and more reliable, when given a short stick, and so we also provided them with a stick to point to the cups during testing. Training sessions, each lasting no more than 5 minutes, were run every day with each subject until they were reliable. This lasted no more than three weeks.

Monkey Haven, Isle of Wight

Background

The Monkey Haven houses the research facilities of the Macaque Cognition project from the University of Portsmouth (researchers involved: Marine Joly, Bridget Waller and Jerome Micheletta).

Animals

15 primate species, with a total of about 60 individuals, lived at the Monkey Haven during the study. Three rhesus macaques belonging to a group of 5 and 3 Barbary macaques belonging to a group of 5 were included in this study.

Enclosures

The monkeys were housed in enriched enclosures, equipped with climbing structures and enrichment devices (food puzzles, boxes, etc.). They all have access to an outdoor compound. They lived in social groups consisting of 4-6 individuals. Furthermore, only those subjects voluntarily entering the area with the experimental setup participated in the study to ensure low stress levels.

Diet

The monkeys were fed daily with assorted fruits and vegetables, nuts, seeds and commercial monkey pellets. They all had access to food and water prior to and during the experiment. Neither food nor water was deprived for the purpose of experiments.

Ethical approval

Animal Welfare and Ethical Review Body (AWERB) of the University of Portsmouth

Research permission

The research received approval by the AWERB (approval no. 4015B). All aspects of the study were covered by this ethical approval.

Research training

All individuals were trained by the research staff from the Macaque Cognition Project, University of Portsmouth, under the protocols approved by the AWERB.

Research participation

Cognitive testing required subjects to break from their social group and enter the testing area voluntarily. Only those subjects voluntarily entering the area with the experimental setup participated in the study to ensure low stress levels.

Other

Monkeys were monitored daily by research and care staff for psychological and physical wellbeing.

Zoo Heidelberg

Background

Zoo Heidelberg was founded in 1933 and currently houses 155 animal species, including gorillas and chimpanzees. The Zoo supports and conducts scientific studies on a regular basis.

Animals

The western lowland gorillas (*Gorilla gorilla gorilla*) housed at Zoo Heidelberg live in a social group of 4 animals. Three of the gorillas (1 male & 2 females) participated in the study, however, data collection could only be completed with the 2 females. The male stopped participating.

Enclosures

The enclosure of the gorillas consists of outdoor (160 m^2) and indoor areas (393 m^2) with connecting tunnels and several smaller compartments to separate the individuals if necessary. The enclosure is equipped with tree trunks, ropes and nets to climb, stone and wooden platforms to sit or lie on. There are plastic barrels, wooden puzzle boxes and large plastic balls available for enrichment. The floor is covered with bark mulch, straw and wood wool. Between the gorilla and chimpanzee enclosure windows allow the apes to see each other and even interact on occasion.

Diet

The gorillas are fed with a variety of vegetables, leaf-eater pellets and browse, additionally some cereals, nuts, puffed rice etc. for enrichment. Water is available ad libitum. Neither food nor water was deprived for the purpose of experiments.

Ethical approval

Non-invasive studies are reviewed and approved by Zoo Heidelberg. This study was approved by the Heidelberg Zoo scientific department, consisting of Dr. Klaus Wünnemann, Director, Sandra Reichler, curator for mammals, conservation and research and Dr. Barbara Bach, zoo veterinarian. The scientific department is under continued supervision of the ethics committee of Heidelberg Zoo, headed by Dr. Klaus Zuber, Director of the Veterinary Department of the city of Heidelberg. We don't use permit numbers, therefore there is no number available for this study. Zoo Heidelberg is accredited by the European Association of Zoos and Aquaria (EAZA) and the World Association of Zoos and Aquariums (WAZA).

Research permission

Research conducted at Zoo Heidelberg complies with international and national standards and laws (e.g. Guidelines for the Treatment of Animals in Behavioural Research and Teaching published by the Association for the Study of Animal Behaviour) and institutional guidelines. Non-invasive studies are reviewed and approved by the curator and veterinarian of Zoo Heidelberg. Further IRB/IAUCUC approval was not necessary because no special permission for the use of animals in purely behavioural or observational studies is required in Germany (TierSchGes §7 and §8).

Research training

An experienced primate researcher working with apes and monkeys for more than 10 years conducted the experiments. In addition, individuals working with the animals are trained by animal caretakers and receive further safety instructions.

Research participation

All subjects participate voluntarily. No participation is ever coerced. Only subjects entering the testing area voluntarily participate in the experiments to ensure no stress is induced. No sliders are closed, and all animals can choose to end the testing and walk away whenever they like.

Other

As the outdoor enclosure of the gorillas is going to be rebuilt soon, no breeding is taking place at the moment.

While the gorillas had experience using touchscreen computers, they had not participated in any manual task before. Therefore, before data collection could begin, we first had to train them to point to a food reward so that they could successfully indicate their selections during test. To do so, we placed a small piece of fruit (e.g. grape or slices of pear) on the testing platform in view of, but out of reach of, a gorilla. When pointing at the food, either with their whole hand, a finger, or stick, they were handed the reward. Training sessions, each lasting no more than 10 minutes, were run every day with each subject until they were reliable.

Sweetwaters Chimpanzee Sanctuary

Background

The Sweetwaters Chimpanzee Sanctuary is located in Kenya. The Sanctuary was established with an agreement between the Ol Pejeta Conservancy, the Kenya Wildlife Service (KWS) and the Jane Goodall Institute. The aim is to provide lifelong refuge to orphaned chimpanzees from West and Central Africa.

Animals

Most of the apes were born in the wild and came to the sanctuary after being confiscated at an early age (~2-3 years old) as a result of the trade in apes for pets and bushmeat. Once the apes arrived at the sanctuary, they were raised by humans together with peers until they were old enough to join a mixed-age social group. Sweetwaters Chimpanzee Sanctuary hosts two groups of chimpanzees (17 individuals in group one, 10 females and 7 males, all between 4 and 31 years of age and 22 individuals in the second group, 10 females and 12 males, all between 1 and 39 years of age). Twenty-one chimpanzees participated in the study (13 from group 1 and 8 from group 2).

Enclosures

All chimpanzees live in social groups. During the day the chimpanzees had access to large tracts of outdoor enclosures, including trees, bushes and climbing structures. The enclosure sizes are: 29.09 hectares (group 1) and 35.31 hectares (group 2). In the evening, all individuals come back from the outdoor enclosures and stay in indoor enclosures overnight. Subjects are tested in familiar rooms in their indoor enclosures.

Diet

Subjects were never food or water deprived for any reason and their diet was supplemented by being fed three times daily with a combination of fruits, vegetables, and other species' appropriate foods.

Ethical approval

The full procedure of the study was approved by the local ethics committee at the Sanctuary (the board members and the veterinarian), the Kenya Wildlife Service and the National Council for Science and Technology, Kenya.

Research permission

The board members and the veterinarian at the Sweetwaters Chimpanzee Sanctuary (Kenya), Kenya Wildlife Service (KWS) and the National Council for Science and Technology, Kenya approved our research. A research permit was issued by the National Council for Science and Technology, Kenya (NACOSTI/P/19/7557/27803; NACOSTI/P/18/24055/20857).

Research training

The study was conducted by researchers with many years of experience with conducting behavioral experimental research with chimpanzees.

Research participation

All testing is strictly voluntary. During testing a subject can indicate her wish to stop participating at any time (e.g. by leaving the test area and / or sitting by the door into another room). All apes are highly motivated to participate.

Other

No breeding program is adopted in the sanctuary.

Kristiansand Zoo

Background

Kristiansand Dyreparken (Zoo) is located 11 km east of Kristiansand (Norway). Kristiansand Zoo is the largest zoological institution in Norway and the only one in the country to house great apes. The institution houses in total eight species of primates.

Animals

Kristiansand Zoo houses a breeding group of 17 ring tailed lemurs and three male black faced spider monkeys. All animals are captive born. Three female lemurs were tested as a group but only the two highest ranking females completed all trials. Only one spider monkey (the dominant male) completed all trials due to monopolization of the set up.

Enclosures

All tests were conducted in the sleeping rooms of the monkeys off-sight from the visitors. The lemurs had access to two indoor enclosures, one outdoor enclosure and a sleeping room approximately 5x3m. The lemurs were housed in two groups to prevent males from attacking the three newborns of the group. The two groups alternated indoor enclosures every day, as only one of the indoor enclosures allowed access to the outdoor enclosure. The spider monkeys had access to three connected sleeping rooms, each approximately 5 to 7m long and 2m wide. They were housed in a single group and had constant access to the indoor and outdoor enclosures and sleeping rooms (except during cleaning hours). Tests took place while the outdoor and indoor enclosures were being cleaned. Once tests and cleaning routines were finished, the subjects had access to both indoor and outdoor enclosures. Both indoor and outdoor enclosures were equipped with structural enrichment such as climbing frames, hose hammocks, artificial trees and logs, and nesting material. In the indoor and outdoor enclosures, the subjects had access to feeders where food was hidden every morning. The outdoor enclosure of the spider monkeys consisted of an island surrounded by a 5m wide filled moat. The island included hanging bridges, huts and climbing frames. The lemurs' outdoor enclosure consisted of a fenced natural nordic forest.

Diet

The animals were never water of food deprived. Diets at the zoo are designed by a veterinarian according to the species nutritional requirements. The lemurs' and spider monkeys' diets consisted of fresh fruits and vegetables, together with lower quantities of primate pellets and nuts. Food was provided twice a day, once in the morning and once in the afternoon. Feeding consisted in the scattering of food in the enclosures as well as the provision of food in localized

areas. The rewards provided during testing were pieces of the subject's favourite fruit already present within the subject's diet.

Ethical approval

The testing methodology for this project was approved by the Ethical Board for Scientific Research at Kristiansand Zoo, lead by Rolf Arne, and by the Kristiansand Zoo Primate Project. The Ethical Board at Kristiansand Zoo does not issue permit numbers. Kristiansand Zoo is a member of the European Association of Zoos and Aquaria (EAZA) and the World Association of Zoos and Aquariums (WAZA).

Research permission

Research permission was granted by the Ethical Board for Scientific Research at Kristiansand Zoo, who internally decides which projects are conducted at the zoo. Decisions within this committee are made in collaboration with the veterinary staff and the animal keepers.

Research training

The study was conducted by a doctoral candidate with experience in designing and performing cognitive tests with different primate species. The experimenter had conducted previous research at the testing institution and was familiar with the husbandry and safety procedures of the zoo.

Research participation

Participation was completely voluntary and was conducted while the subjects were in their sleeping rooms as an enrichment activity. The only measure taken during the tests was to call the subjects' names if the subjects stopped participating to regain their attention.

Other

The lemurs included in this study are part of a breeding group and had infants with them (three) during the time of testing. Females with infants were kept separated from the rest of the group to prevent aggression from the males.

German Primate Center

Background

The German Primate Center is a research institute studying primates and currently houses 6 species of primates.

Animals

Lemurs: The ring-tailed and black-and-white ruffed lemurs were born in captivity and are housed in enriched outdoor and indoor cages at the German Primate Center. We tested 7 out of 8 ring-tailed lemurs belonging to two groups with 3 and 5 individuals. We tested 7 black-and-white ruffed lemurs that live in one group.

Macaques: The Cognitive Ethology Lab of the German Primate Center contributed data of 17 long-tailed macaques (*Macaca fascicularis*) to this study. The monkeys were born in captivity and live in a social group of 36 individuals at the time of data collection (29 females, 7 males; age range: 1 - 30 years).

Enclosures

Ring-tailed lemurs are housed in indoor enclosures with one room of 3x3m and two rooms of 3x3m for each group and an outdoor enclosure of 29x20m. The black-and-white ruffed lemurs are housed in two indoor enclosure of 4.4x3.6m and an outdoor enclosure of 29x20m. The two outdoor enclosures are next to each other, so that the two species see each other. All enclosures are enriched with tree trunks, ropes and nets to climb, as well as wooden platforms to sit or lie on.

The long-tailed macaques have access to indoor and outdoor enclosures (49m^2 and 141m^2 respectively), which are equipped with various enrichment objects, wooden platforms, fire hoses, and a water basin during the warm months.

Diet

Lemurs: Both species are fed with a variety of vegetables, fruits and monkey chow. Water is available ad libitum. Neither food nor water was deprived for the purpose of experiments

Long-tailed macaques: The macaques were not food or water deprived for testing and were fed their normal diet of monkey chow, fruits and vegetables twice a day. Water was available ad libitum.

Ethical approval

Lemurs and long-tailed macaques: Animal Welfare body of the German Primate Center.

Research permission

Lemurs: Non-invasive studies have been reviewed and approved by the Niedersächsische Landesamt für Verbraucherschutz und Lebensmittelsicherheit and the Animal Welfare body of the German Primate Center.

Long-tailed macaques: The experiment was conducted in accordance with the German Animal Welfare Act and were approved by the ethics committee of the Animal Welfare Body of the German Primate Center (Permit Numbers: Lemurs E3-18_4-17, Long-tailed macaques: E3-18_9-17). Permission of the Lower Saxony State Office for Consumer Protection and Food Safety was not required (LAVES Document 33.19-42502-04).

Research training

Lemurs: Animals are trained by experienced animal caretakers and technical assistants. The experiments of this study were conducted by an experienced primate researcher working with lemurs for more than 20 years and a technical assistant.

Long-tailed macaques: The researchers were trained to work with the respective populations according to the local safety instructions. The monkeys were familiar with the researchers and were used to interact with them for the purpose of training and testing.

Research participation

Lemurs: All subjects participate voluntarily in the experiments and were never coerced. Subjects voluntarily entered the indoor testing cage, which is part of their home cage and could choose to end the testing by walking to the door, which then immediately opened.

Long-tailed macaques: The monkeys are tested in a testing area adjacent to their indoor enclosure. The researchers offer participation in experiments by opening the doors between indoor enclosure and test area and monkeys are never forced to enter if they do not want to. During testing, the doors between indoor enclosure and test area are closed; the monkeys are used to be separated from their group for short periods of time when they participate in cognitive experiments. During testing, the subjects remain in visual and auditory contact with their group.

Other

Lemurs: Adult females of both species are involved in a breeding program.

Ape Cognition and Conservation Initiative

Background

The Ape Cognition and Conservation Initiative was formed in 2013 under the direction of Dr. William Hopkins and Dr. Jared Taglialatela. The facility is located on 230 acres of land outside of Des Moines, Iowa and currently houses five bonobos (*Pan paniscus*). Researchers from across the globe collaborate with ACCI staff and scientists to conduct research on great ape communication and cognition.

Animals

ACCI contributed data from five bonobos. Four males: Kanzi (38), Maisha (18), Teco (8), and Nyota (20). One female: Elikya (21). All five bonobos have lived at the facility since 2005.

Enclosures

The bonobos at ACCI are housed in 13 different indoor enclosures ranging in size from roughly 500 square feet to 1350 square feet. All enclosures are equipped with environmental enrichment and allow for both research and enrichment apparatuses to be added and removed. Bonobos can willingly separate into any of these spaces to participate in cognitive research, or socialize in groups of 2 or more for behavioral research. There are two outdoor yards with 6 acres of ape space where researchers can observe the apes from a birds-eye view. ACCI has roughly 2815 square feet of human-only areas, including office space, a public lobby, a kitchen area for preparing food for the apes, and a vet suite.

Diet

Animals are maintained on a veterinarian approved diet consisting of various fruits, vegetables, seeds, and nuts provided throughout each day in meals, foraging enrichment, and as rewards during cognitive testing. The bonobos are never water restricted.

Ethical approval

The research was approved by the IACUC committee of ACCI (Protocol #170904-01R), no permit number issued. ACCI is certified by the Association of Zoos and Aquariums.

Research permission

All research performed at ACCI is approved by ACCI's IACUC committee. Researchers must be listed on an approved IACUC document.

Research training

All researchers and staff at ACCI have completed rigorous online and in-person training to safely work around apes. All visiting researchers have completed online and in-person training and are required to remain at least 3 feet away from animal enclosures during testing.

Research participation

All individuals participate in cognitive testing by voluntarily entering testing spaces and willingly separating themselves from other individuals, when necessary.

Other

There is no breeding program at place at ACCI. In the future, ACCI plans to introduce and house additional bonobos from various AZA-accredited facilities across the United States. After introduction into stable social groups, ACCI plans to include these individuals in future research programs.

Living Links, RZSS Edinburgh Zoo

Background

The 'Living Links to Human Evolution' Research Centre in RZSS Edinburgh Zoo has been designed as a scientific institution managed in collaboration between the Royal Zoological Society of Scotland, the University of St Andrews and the Scottish Primate Research Group, which represents a consortium of primatologists at a number of Scottish Universities. The Centre has been created to facilitate behavioural, cognitive and welfare-based research on naturalistically housed monkeys, at the same time introducing the zoo-visiting public to the science embodied in these enterprises, in an educational and even entertaining way.

Enclosures

Enclosures for the two mixed species groups mirror each other on either side of a central viewing platform, and are named the 'West' and 'East' wings. Each wing includes an indoor squirrel monkey enclosure (5.5m by 4.5m by 6m high), to which only the squirrel monkeys have access, an indoor capuchin enclosure to which both species have access (7m by 4.5m by 6m high), and a large shared outdoor enclosure (approximately $900m^2$) to which both species have access. Between each pair of inner monkey enclosures is a research room, along each side of which is a set of two banks of cubicles, which form an entry and exit route for the monkeys, between their inner and outer enclosure. These cubicles can either be opened up to each other, or separated by transparent or opaque slides, thus providing a highly flexible research environment. Individual cubicles are $0.5m^3$, providing a run of 2m long and 1m high for each entire bank. The monkeys have permanent access to all areas of their enclosure except in inclement weather.

Animals

The Centre has housed two mixed species communities of common squirrel monkeys (*Saimiri sciureus*) and brown (tufted) capuchin monkeys (*Sapajus sp.*). These species co-habit in the wild. In the wild capuchins have been shown to have a relatively small home range of $0.8km^2$ and squirrel monkeys about $2km^2$. There are 35 capuchin monkeys (18 West, 17 East) and 30 squirrel monkeys (13 West, 17 East as of November 2015). The two species live well together (Buchanan-Smith et al., 2013).

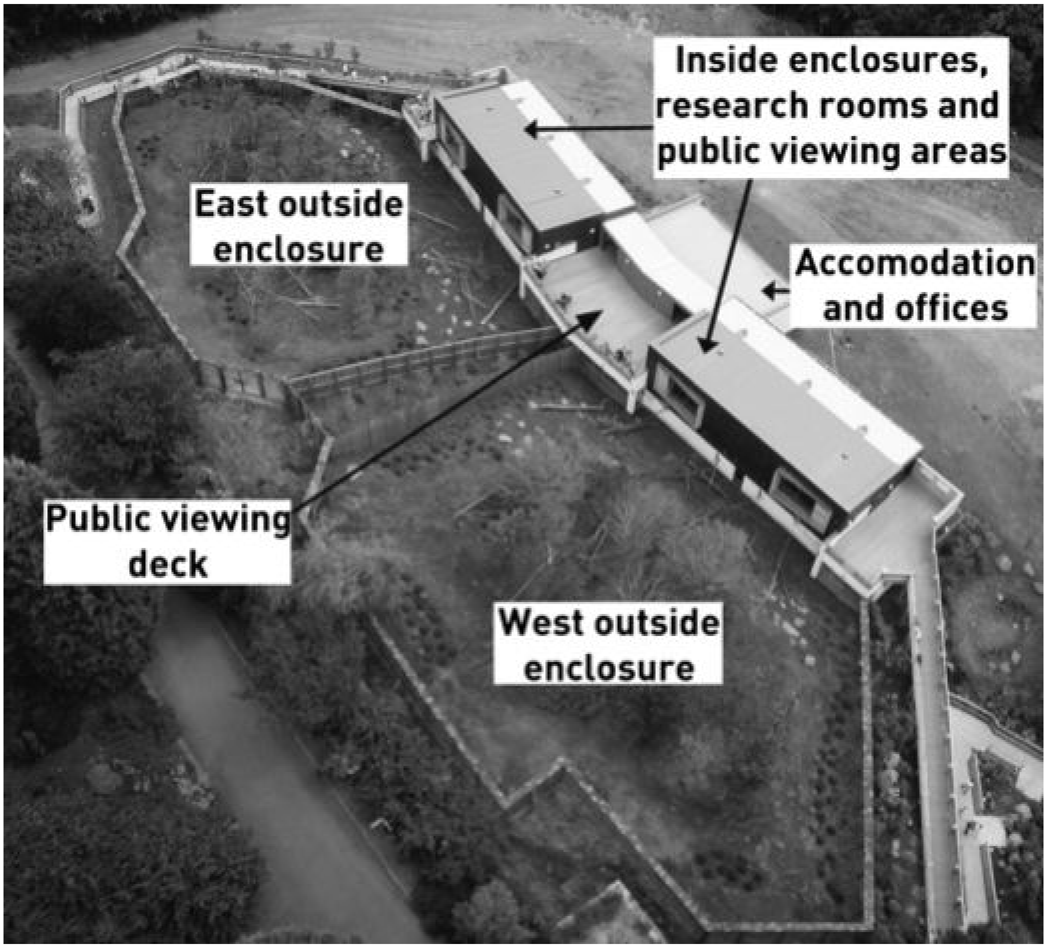

Diet

The monkeys are fed on a rich diet of meat, eggs, vegetables, fruit and monkey cereals. They are fed four times a day as well as receiving regular food through enrichment devices and research rewards. The monkeys are not food or water deprived in their enclosures and never food or water deprived before research. See below for research procedures relating to food.

Ethical approval

The research was approved by School of Psychology & Neuroscience Ethics Committee of the University of St Andrews (project entitled “Working memory in new world monkeys and great apes” was approved on 10/04/2018; no permit number was issued).

Research permission

All projects must be approved by the research liaison officer (Living Links Team Leader employed by the zoo), the research director (employed by the University of St Andrews) and a Research Fellow based at the zoo (employed by an SPRG member university). Research projects

must have ethical approval from the lead researcher's institution and all researcher's involved in the project need a Basic Disclosure Scotland before they can work at the zoo.

Research training

All researchers undergo a relevant induction and training. This occurs after the project has received zoo approval and before the study begins. This may or may not be done before university ethical approval. However, studies cannot begin until the zoo receives evidence of university ethical approval. Training is led by a senior keeper. On their first day in the facility, researchers receive training relating to zoo Health and Safety, Zoo policies and theoretical training pertinent to their project. This is all based on an induction handbook which is emailed to researchers. Those working in the research rooms are then given additional practical training from a trained keeper. This involves about eight to 24 sessions (depending on training criterion being reached) where the keeper and the researcher work in the research rooms together. Researchers are trained by keepers to recognise individual monkeys, operate the sliders safely and identify behaviours in the monkeys. They are also given training on escapes and emergencies. Only once the keepers are satisfied that the researchers can work safely and can react appropriately to the animals' cues are they able to begin their study. Researchers also have an ID test with a keeper to ensure they know the identity of the monkeys.

Research participation

Most monkeys have been habituated to remain in the research cubicles for research sessions in which they may be either by themselves or in various social configurations required for the particular research question under consideration. Participation is voluntary. A monkey is never forced to come into the research cubicles. Monkeys are isolated for up to 15 minutes, up to twice a day, four days a week. There is a clock in each research room and stop watches available to assist with time keeping. If the monkeys show any signs of distress they are reintroduced to the group immediately. These signs of distress include ceasing participation, moving to the back of the cubicle and/or putting hands on the cubicle slides and/ or specific vocalisations.

There is no access to ad-libitum food and water in the cubicles but monkeys are given regular food rewards during all research. The monkeys may be rewarded with sunflower seeds, nuts, raisins, dates, cereal and mealworms. There are maximum allowances for these which have been decided by senior members of the husbandry team.

Other

The West squirrel monkey group is a breeding group, the other three populations (East squirrel and East and West capuchin groups) are not breeding. Infants tend to stay on their mothers until they are about a year old. During this time the mother can still participate in research if they seem comfortable. Infants will not be isolated until there is an assessment by the keepers that the

infants are regularly ‘off’ of their mothers and both the infant and the mother show no signs of distress during isolation.

References

Buchanan-Smith,Hannah M. Griciute, Joana, Daoudi,Sophia, Leonardi, Rebecca and Whiten, Andrew (2013). Interspecific interactions and welfare implications in mixed species communities of capuchin (*Sapajus apella*) and squirrel monkeys (*Saimiri sciureus*) over 3 years. *Applied Animal Behaviour Science.*

Macdonald, C. & Whiten, A. (2011). The ‘Living Links to Human Evolution’ Research Centre in Edinburgh Zoo: a new endeavour in collaboration. *International Zoo Yearbook*. Volume 45, Issue 1, pages 7–17.

Budongo Research Unit, RZSS Edinburgh Zoo

Background

The ‘Budongo Research Unit’ in RZSS Edinburgh Zoo is a research facility of the University of St Andrews in collaboration with the Royal Zoological Society of Scotland. Members of the Scottish Primate Research Group, a consortium of primatologists in several Scottish Universities, also participate in this collaboration. The Unit was created to promote the advancement of the scientific knowledge of the behavior, cognition and welfare on naturalistically housed chimpanzees. It provides a unique opportunity to zoo visitors to observe researchers at work and learn about the latest developments in this area.

Enclosures

Enclosures include a large outdoor area (1985 m^2), three indoor ‘pods’ (total floor area 309 m^2, approx. 10m traversable height in each), and an off-view ‘beds’ area (55 m^2), all interconnected by a tunnel system (30m^2 approx.), and with access to climbing structures mounted on natural substrate (e.g. grass, dirt). Indoor pods provide varying levels of natural light, and are air conditioned and temperature monitored. Moreover, enclosures possess visual barriers and an off-view area that allow individuals to retreat from other group members or the zoo visitors. Enclosures have access to free-flowing water and include a number of food enrichment devices provided to individuals daily. The group has access to all indoor areas during the night, which have multiple raised sleeping platforms at varying heights, and are provided with natural bedding materials (e.g., eucalyptus leaves, wood wool) and additional blankets/cardboard from which to make their nests. The main research area (30 m^2, 2.12m high) is adjacent to the chimpanzee indoor enclosures. It consists of three adjoining research rooms that can be used as one large research area or split into three smaller research areas by hydraulic doors. Access to the research area and participation in research activities is completely voluntary. Moreover, there are multiple access routes from the indoor pods and tunnel system into the research area so that chimpanzees can enter and exit from multiple directions and never feel trapped in by other individuals of their group.

Animals

The Unit houses a group of 17 chimpanzees (*Pan troglodytes*), seven adult males, nine adult females and one juvenile male.

Diet

The chimpanzees are fed on a varied diet of vegetables, fruit, nuts, seeds, eggs and vegetation browse. They are fed four times a day as well as receiving regular food through enrichment

devices and research rewards. The chimpanzees are not food or water deprived in their enclosures and never food or water deprived before research. See below for research procedures relating to food.

Ethical approval

The research was approved by School of Psychology & Neuroscience Ethics Committee of the University of St Andrews (project entitled "Working memory in new world monkeys and great apes" was approved on 10/04/2018; no permit number was issued).

Research permission

All projects must be approved by the research liaison officer (Living Links Team Leader employed by the zoo), the research director (employed by the University of York) and a Research Coordinator based at the zoo (employed by the University of St Andrews). Research projects must have ethical approval from the lead researcher's institution and all researchers involved in the project need a Basic Disclosure Scotland before they can work at the zoo.

Research training

On their first day in the facility, researchers receive training on health and safety as well as testing procedures. This information is compiled in the induction handbook that the research coordinator sends to each inductee. Once the project has been approved by the Zoo and the University ethics committee, the project may begin. Researchers are accompanied at all times by a keeper who provides practical advice and support during testing. Senior researchers may be approved to work on their own after a period of testing under keeper supervision.

Research participation

Most chimpanzees enter the research area and participate in our research sessions. They may enter as a group of varying composition or individually. Participation is strictly voluntary and they are free to leave the area at any time. If the chimpanzees show signs of distress (e.g., whimpering) during the test, we terminate it immediately. Chimpanzees receive food and/or fruit juice (diluted in water) during tests. Solid food includes apples, raisins, cereal and grapes. There are maximum food allowances for each of these items set by the senior members of the husbandry team.

Other

Wolfgang Köhler Primate Research Center, Leipzig, Germany

Background

The Wolfgang Köhler Primate Research Center (WKPRC) is a project of the Max Planck Institute for Evolutionary Anthropology. It is operated in collaboration with the Leipzig Zoo. Research focuses on the behavior and cognition of the four species of great ape: chimpanzees (*Pan troglodytes*), gorillas (*Gorilla gorilla*), orangutans (*Pongo pygmaeus*), and bonobos (*Pan paniscus*). Researchers and students from the University of Leipzig, and other universities around the world, conduct their research projects at the center guided by the personnel of the Center.

Animals

Eleven chimpanzees and 5 orangutans participated in this study. The chimpanzees sample consisted of 7 females (age range 16-42) and 4 males (age range 14-43); the orangutan sample consisted of 4 females (15-30) and one male (38). All individuals live in social groups.

Enclosures

The indoor space featured climbing structures, wooden platforms, visual barriers. The outdoor space had a grass floor, climbing trees, wooden platforms and visual barriers. They had access to their outdoor exhibit area whenever weather permitted. The total size of the indoor/outdoor enclosure was 430/4000 m^2 for the chimpanzees and 230/1680 m^2 for the orangutans. Both enclosures are equipped with shaking boxes and poking bins which allow the apes to engage in activities similar to their natural social and foraging behaviors (e.g., tool use).

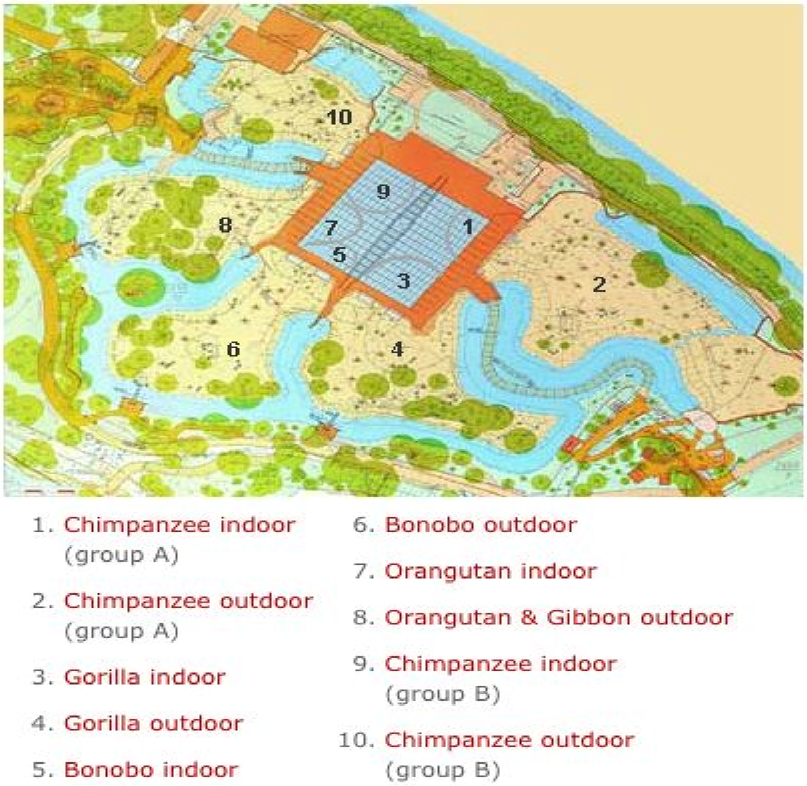

Diet

The chimpanzees and orangutans were fed with a variety of fresh fruit and vegetables daily. The food rewards that were used for this study (grapes) were reviewed and approved by veterinary and nutrition staff prior to the start of the experiment. In addition, apes regularly receive special foodstuffs (e.g., chestnuts) that the keepers hide in certain areas of the enclosure to promote natural foraging activities. Other opportunities for special foraging activities (e.g., artificial termite mounds) are also made available on a regular basis and special enrichment materials are provided for the apes every afternoon. Subjects are never deprived of food or water.

Ethical approval

The study was ethically approved by the internal ethics committee of the Max Planck Institute for Evolutionary Anthropology and Leipzig zoo. Members of the committee are: director of the WKPRC Dr. J. Call, research coordinator at WKPRC Dr. D. Hanus, zoo veterinarian Dr. A. Bernhard, head animal keeper F. Schellhardt and assistant head animal keeper M. Lohse. All research done at WKPRC is approved by this committee. No medical, toxicological or neurobiological research of any kind is conducted at the WKPRC. Research was non-invasive and strictly adhered to the legal requirements of Germany. Animal husbandry and research

comply with the “EAZA Minimum Standards for the Accommodation and Care of Animals in Zoos and Aquaria”, the “WAZA Ethical Guidelines for the Conduct of Research on Animals by Zoos and Aquariums” and the “Guidelines for the Treatment of Animals in Behavioral Research and Teaching” of the Association for the Study of Animal Behavior (ASAB).

Research permission

Permission was given by an internal committee of the Max Planck Institute for Evolutionary Anthropology and the Leipzig Zoo (see above). No permit number was issued. Further IRB/IAUCUC approval was not necessary because no special permission for the use of animals in purely behavioural or observational studies is required in Germany (TierSchGes §7 and §8).

Research training

The researchers are all employees of the Max Planck Institute. The keepers are all employees of Leipzig Zoo and so have received extensive training working with these animals.

Research participation

All individuals were tested individually. Only those subjects voluntarily entering the area with the experimental setup participated in the study and a session would be stopped if a subject showed any sign of distress.

Other

In cooperation with the zoo, the Köhler Center supports efforts to conserve great apes, both in the wild and in captivity. The breeding program at the zoo is framed within the global strategy of the European Endangered Species Program (EEP), and some research focuses on the husbandry and care of great apes in captivity.

Acknowledgements

For data collected at Edinburgh Zoo, we thank Abi Gwynn for her help with reliability coding.

We thank Charlotte Gurney-Read for helping collecting and coding the data and the Monkey Haven, Isle of Wight.

For the works at Kumamoto Sanctuary, we thank S. Hirata, N. Morimura, R. Song for their help in data collection and coding, T. Udono, M. Teramoto, H. Ogi, and E. Nogami for their husbandry efforts.

For data collected at Lincoln Park Zoo, we thank Jill Moyse and Danielle Fogarty for their assistance, and to the Regenstein Center for African Apes animal care staff for facilitating our research and providing the best possible care to the apes. We also thank the Chauncey and Marion Deering McCormick Foundation and the Leo S. Guthman Fund for their support of this research.

The Swiss National Science Foundation provided funding to Manon K. Schweinfurth (grant number P2BEP3175269).

For data collection at the Sweetwaters Chimpanzee Sanctuary, we thank Richard Vigne, Stephen Ngulu, the board members and all the staff of Sweetwaters Chimpanzee Sanctuary in Kenya for their crucial support during all stages of this research. We also thank Ol Pejeta Conservancy, Kenya Wildlife Service (KWS), and the National Council for Science and Technology (NCST) for approving our research.

For data collection at Kristiansand Zoo we are grateful to Tanya Michin and Helene Axelsen for assistance with the project. AMR is also grateful to the STONECULT project for economic support during data collection.

For data collection at the Language Research Center at Georgia State University, we thank Myah Vogt as well as the animal care staff.

For data collection at the Ape Cognition and Conservation Initiative, we thank Amanda Epping, Jared Taglialatela, William Hopkins, and the animal care staff for their support and assistance in data collection for this project.

Manuel Bohn received funding from the European Union's Horizon 2020 research and innovation programme under the Marie Sklodowska-Curie grant agreement no. 749229.
